# A levy chaotic horizontal vertical crossover based artificial hummingbird algorithm for precise PEMFC parameter estimation

**DOI:** 10.1038/s41598-024-81168-6

**Published:** 2024-11-28

**Authors:** Pradeep Jangir, Absalom E. Ezugwu, Kashif Saleem, Arpita, Sunilkumar P. Agrawal, Sundaram B. Pandya, Anil Parmar, G. Gulothungan, Laith Abualigah

**Affiliations:** 1 University Centre for Research and Development, Chandigarh University, Gharuan, Mohali 140413 India; 2 Department of CSE, Graphic Era Hill University, Dehradun, 248002 India; 3 Department of CSE, Graphic Era Deemed To Be University, Dehradun, 248002 Uttarakhand India; 4 Applied Science Research Center, Applied Science Private University, Amman, 11931 Jordan; 5 Unit for Data Science and Computing, North-West University, 11, Hofman Street, Potchefstroom, 2520 South Africa; 6 Department of Computer Science and Engineering, College of Applied Studies and Community Service, King Saud University, 11362 Riyadh, Saudi Arabia; 7 Department of Biosciences, Saveetha School of Engineering, Saveetha Institute of Medical and Technical Sciences, Chennai, 602 105 India; 8 Department of Electrical Engineering, Government Engineering College, Gandhinagar, Gujarat 382028 India; 9 Department of Electrical Engineering, Shri K.J. Polytechnic, Bharuch, 392 001 India; 10 Department of Electronics and Communication Engineering, SRM Institute of Science and Technology, SRM Nagar, Kattankulathur, Chengalpattu, Tamilnadu 603203 India; 11 Computer Science Department, Al Al-Bayt University, Mafraq, 25113 Jordan; 12 Centre for Research Impact & Outcome, Chitkara University Institute of Engineering and Technology, Chitkara University, Rajpura, 140401, Punjab, India; 13 Jadara University Research Center, Jadara University, PO Box 733, Irbid, Jordan

**Keywords:** PEM fuel cell, Optimal parameter estimation, Electrical Engineering Optimization, Artificial hummingbird algorithm, LCAHA, Electrochemistry, Energy, Chemical engineering, Electrical and electronic engineering

## Abstract

In this research, enhanced versions of the Artificial Hummingbird Algorithm are used to accurately identify unknown parameters in Proton Exchange Membrane Fuel Cell (PEMFC) models. In particular, we propose a multi strategy variant, the Lévy Chaotic Artificial Hummingbird Algorithm (LCAHA), which combines sinusoidal chaotic mapping, Lévy flights and a new cross update foraging strategy. The combination of this method with PEMFC parameters results in a significantly improved performance compared to traditional methods, such as Particle Swarm Optimization (PSO), Differential Evolution (DE), Grey Wolf Optimizer (GWO), and Sparrow Search Algorithm (SSA), which we use as baselines to validate PEMFC parameters. The quantitative results demonstrate that LCAHA attains a minimum Sum of Squared Errors (SSE) of 0.0254 and standard deviation of 4.59E−08 for the BCS 500W PEMFC model, which is much lower than the SSE values obtained for PSO (0.1924) and GWO (0.0364), thereby validating the superior accuracy and stability of LCAHA. Moreover, LCAHA converges faster than DE and SSA, reducing runtime by about 47%. The robustness and reliability of LCAHA-simulated and actual I–V curves across six PEMFC stacks are shown to be in close alignment.

## Introduction

DC microgrids are increasingly becoming more efficient and reliable, primarily due to the prevalence of DC loads and the DC output from various sources like renewable energy, storage systems, and fuel cells. Fuel cell (FC) systems are integral components within DC microgrids. The combination of solar energy with hydrogen, known for being a safe and sustainable storage system, forms the basis of hydrogen energy^1,2^. Although hydrogen is the third most abundant element on Earth, found in water, fossil fuels, and other minute entities, after oxygen and silicon^3,4^, hydrogen gas does not naturally exist in isolation, except within natural gas reservoirs^5^. This resource is garnering increasing global interest^6^.

Fuel cells are electrochemical devices that convert the chemical energy of hydrogen into electricity^7,8^. They are becoming increasingly popular across transportation, portable, and stationary applications due to their significant benefits such as high efficiency, clean and quiet operation, and high power and energy density^9,10^. Presently, the market features several types of fuel cells, with the most notable being proton exchange membrane FC (PEMFC)^11^, alkaline FC (AFC)^12^, solid oxide FC (SOFC)^13^, phosphoric acid fuel cell (PAFC)^14,15^, and microbial fuel cell (MFC)^16,17^.

Mann’s model is one of the semi-empirical models used to describe PEMFC performance, which consists of seven unknown parameters^18^. Accurately determining these parameters is crucial as the model’s precision is dependent on them. These parameters can be extracted using either meta-heuristic optimization algorithms or traditional analytical methods. Among the conventional methods are the stochastic method^19^, the input–output diffusive approach^20^, and the proper generalized decomposition approach^21^. Geem and colleagues^22^ employed the generalized reduced gradient method. However, these methods are often limited by their reliance on the initial conditions of the problem, the risk of converging to a local minimum, and their accuracy being contingent on the error of the differential equations’ solver^5^.

Given the limitations discussed earlier, numerous researchers have turned to meta-heuristic algorithms due to their flexibility with problem formulations, derivative-free nature, and applicability to diverse real-world engineering challenges^23^. Specific researchers have applied unique algorithms; EL-Fergany and colleagues adopted the grasshopper optimizer^16^, whale optimization algorithm^17^, and salp swarm optimizer^24^. Seleem and associates used the equilibrium optimizer in their research^25^, while Alsaidan applied the chaos game optimization technique^26^. Sultan and his team identified fuel cell parameters using improved chaotic electromagnetic field optimization^27^, and the artificial ecosystem optimizer was employed in another study^28^. Rao and colleagues used a shark smell optimizer for the PEMFC model^29^, Fahim and associates implemented the hunger games search algorithm^30^, and a novel circle search algorithm was explored in another study^31^.

Additionally, Ali and others proposed using a grey wolf optimizer (GWO) to achieve optimal PEMFC parameters^32^, Abaza and colleagues introduced a coyote optimization algorithm (COA) for solving the PEMFC problem^33^, and Zaki and associates utilized marine predators and political optimizers^34^. Chen and his team implemented a cuckoo search algorithm (CS)^35^, while Kandidayeni and colleagues employed both the firefly optimization algorithm (FOA) and shuffled frog leaping algorithm (SFLA) to model the PEMFC^36^, biogeography-based optimization algorithm (BBO) by Niu et al.^37^, and backtracking search algorithm (BSA) by Askarzadeh^38^, bird mating optimizer (BMO)^39^ and grouping-based global harmony search algorithm (GGHS)^40^. Chakraborty et al. applied differential evolution (DE) to find PEMFC parameters^41^, Priya et al., used flower pollination algorithm (FPA)^42^, Outeiro et al. employed simulated annealing optimization algorithm (SA)^43^.

The computational steps in many of the algorithms discussed earlier are lengthy and involve complex procedure^44^. Also, the nonlinearity characteristics of Proton Exchange Membrane Fuel Cells (PEMFC) make it difficult for a majority of meta-heuristic algorithms hence leading to several limitations. For instance, some of these features lead to premature convergence issues in some algorithms such as Cuckoo Search (CS)^35^, Firefly Optimization Algorithm (FOA)^36^ and Biogeography Based Optimization (BBO)^37^. On the other hand, Grey Wolf Optimizer (GWO)^32^, Shuffled Frog Leaping Algorithm(SFLA)^36^, Backtracking Search Algorithm(BSA)^38^ and Differential Evolution(DE)^41^ suffer from slow convergence rates.

In addition, there exist techniques that need intricate parameter settings and extensive tuning like Flower Pollination Algorithm(FPA)^42^, Bird Mating Optimizer(BMO)^39^,Simulated Annealing(SA)^43^. Also, there is a problem with approaches like CS^35^ and Grouping-Based Global Harmony Search (GGHS)^40^ which easily get trapped at local optimum solutions. Despite these problems, there are notable advantages. For example, The Grey Wolf Optimizer (GWO) as well as Coyote Optimization Algorithm (COA), are known for their simple variable tuning. Additionally, Jellyfish Search Optimizer, Shark Smell Optimizer and Neural Network Optimizer have low computational demands. Furthermore, rapid convergence speeds distinguish COA^45^, Marine Predator Optimizer (MPO) and Equilibrium optimizer (EO). Chaotic Slime Mold Algorithm (CSMA) has been successfully used for multi-disciplinary design optimization problems using chaotic sequences to enhance convergence and exploration capabilities^46^. The Improved Chaotic Harris Hawks Optimizer (ICHHO) also uses chaotic maps to avoid local optima and can be applied to complex numerical and engineering optimization tasks^47^. Furthermore, the Chaotic Slime Mould Optimizer (CSMO) is used to solve the unit commitment problem in an integrated power system with wind and electric vehicles, with the aid of chaos to improve search efficiency^48^. Wan et al. (2023) proposed an analysis method for optimizing water management in PEM fuel cells by examining different operating conditions to achieve optimal hydration states^49^. Zhang et al. (2023) introduced a multiple learning neural network algorithm to improve the accuracy of PEM fuel cell parameter estimation, offering a robust approach for model fidelity^50^. Furthermore, Waseem et al. (2023) reviewed the integration of fuel cells into hybrid electric vehicles, discussing critical challenges, policy implications, and future research opportunities^51^. Lastly, Qiu et al. (2023) outlined progress and identified challenges in multi-stack fuel cell systems for high-power applications, particularly focusing on energy management strategies^52^. A modified manta ray foraging optimization method has been demonstrated to improve parameter identification in PEMFC systems^53^ with better accuracy and stability. A recent study also introduced a modified slime mold algorithm for hydrogen powered PEMFCs, which showed significant improvements in terms of accuracy and convergence^54^. Additionally, the chaotic Rao optimization algorithm has been successfully utilized for steady state and dynamic characterization of PEMFC models, yielding useful information on the reliability and performance of PEMFC stacks under different conditions^55^.

Start with including some more recent studies from the last 3 to 5 years on optimization algorithms in PEMFC modeling. One of these would be references to later metaheuristic and hybrid approaches to Energy Systems for faster convergence speed, better solution accuracy, and stability. For example, studies of algorithms such as Mayfly Optimization Algorithm, Marine Predator Algorithm, Chaotic Harris Hawks Optimization, and others indicate that chaotic maps and adaptive strategies improve algorithm performance. However, these references could point out that, though these methods have succeeded in solving some optimization problems, there are difficulties with these complex engineering problems, such as PEMFCs.

They clearly outline the complex, nonlinear nature of PEMFC systems. The parameters of PEMFCs are interdependent, and include activation, ohmic, and concentration losses, which are different under different operating conditions. The dynamic behavior of PEMFCs under varying loads, pressures and temperatures, as well as the non-linear I-V relationship, make accurate parameter estimation difficult. This complexity, however, poses a challenge to traditional methods such as gradient based techniques or simple metaheuristics, which can suffer from premature convergence or entrapment in local optima, particularly when the search space is high dimensional and multi modal. Note that while such progress has been made, current algorithms are still lacking in directly balancing exploration (globally exploring a solution space) and exploitation (refining a solution with local refinements). A number of algorithms either do not have adequate global search capability and hence converge prematurely or have low convergence rates due to poor local search. For instance, the Grey Wolf Optimizer (GWO) and Differential Evolution (DE) algorithms may convergence slowly or need many parameter tuning, thus may be less practical for PEMFC applications that require real time control.

Artificial Hummingbird Algorithm (AHA)^56^ was selected as the primary algorithm for enhancement because of its unique adaptive mechanisms that match well with the complex and nonlinear nature of PEMFC parameter estimation. AHA is shown to have strong exploration and exploitation abilities, with an intrinsic multi-dimensional search approach based on the foraging behavior of hummingbirds. This enables efficient search space navigation, which prevents entrapment of the search in local optima and encourages global search capabilities. In addition, its framework is flexible to include more sophisticated strategies, e.g., sinusoidal chaotic mapping and Lévy flight, to enhance the convergence speed and accuracy. Although other algorithms exist, AHA structure lends itself naturally to the implementation of these improvements, and is therefore particularly well suited to the precise, time critical applications, such as PEMFC parameter estimation. In this study, the limitations of these algorithms are addressed by the development of the Lévy Chaotic Artificial Hummingbird Algorithm (LCAHA). LCAHA uses chaotic maps and Lévy flights, and couples them with cross update foraging strategies for both exploration and exploitation phases, thus increasing the likelihood of reaching a global optimal solution in a reasonable time. The chaotic map is used to start a random search; the Lévy flight searches globally to escape any local optima, and the cross-update foraging procures quick convergence. For PEMFC parameter estimation, precision and computational efficiency are particularly important, and this is especially so. The research conducted thus fulfills the need for a more robust, adaptive, and efficient optimization method, which LCAHA^57^ is shown to be a suitable solution to the complex optimization problems posed by PEMFC models. Furthermore, this paper provides a thorough comparison with existing methods in the literature to validate and verify the efficacy of these techniques. The following are some insights of the study:

1. The precise extraction of unknown PEMFC model parameters by minimizing the sum of squared errors between measured and simulated data.
2. Development of an accurate PEMFC model that replicates the electrical and electrochemical characteristics of actual PEMFC stacks, considering variations in pressure and temperature of the reactants.
3. An extensive comparative analysis of five optimization algorithms: LCAHA, Particle Swarm Optimization (PSO)^58^, Differential Evolution (DE)^59^, Grey Wolf Optimizer (GWO)^60^, and Sparrow Search Algorithm (SSA)^61^ for parameter extraction in PEMFC models.
4. Evaluation of the efficiency of the applied algorithms using six different PEMFC stacks: BCS 500W-PEM^62^, 500W-SR-12PEM^63^, Nedstack PS6^63^, 12 W-HR-12 PEM^64^, 500WHORIZON PEM^64^, and 250W-stack^65^.
5. Presentation of comprehensive statistical analysis to validate the reliability of the applied algorithms.
6. Comparison of the results obtained from the proposed algorithm with those from various recent algorithms reported in the literature.
7. A competitive comparison highlighting the reliability of the applied algorithms in addressing the studied problem.
8. The main innovation of this study is the Lévy Chaotic Artificial Hummingbird Algorithm (LCAHA), a new multi strategy optimization method that combines sinusoidal chaotic mapping, Lévy flights and an advanced cross update foraging strategy. The integration of this method enhances both exploration and exploitation capabilities, and leads to a significant improvement in the accuracy and convergence speed of parameter estimation for PEMFC models as compared to existing methods.

The remainder of this paper is organized as follows: section "PEMFC Mathematical Modelling" outlines the mathematical model of PEMFC stacks and the objective function. Section "Enhanced artificial hummingbird algorithm" describes the optimization algorithms used. Section "Result Analysis and Discussion" presents the simulation results and dynamic performance of PEMFCs for each case study, along with a statistical analysis. Finally, section "Conclusion" draws the main conclusions of the research.

## PEMFC mathematical modelling

In this section, we first provide a comprehensive description of the semi-empirical model and the specifications of the chosen Proton Exchange Membrane Fuel Cell (PEMFC). Following that, we define the objective function and discuss statistical comparison metrics, including Mean Biased Error (MBE) and the efficiency of the objective function.

### Semi-empirical electrochemical model

The output voltage of the FC stack ($${V}_{fc})$$ is obtained using Eq. (1),

1 $$V_{fc} = \left( {V_{Nernst} - V_{act} - V_{ohmic} - V_{con} } \right) \cdot N_{cell}$$

In this description, ${V}_{act}$​ denotes the activation polarization caused by the slow reaction rates at the electrode surface, ${V}_{ohmic}$​ refers to the ohmic polarization which accounts for the resistance encompassing all electrical and ionic conduction losses through the electrolyte, catalyst layers, cell interconnects, and contacts. ${V}_{con}$​ indicates the concentration polarization linked to the variance in concentration between the fuel/air channel and the chemical species on the electrode surface, and ${N}_{cell}$ is the number of cells^66^. ${V}_{Nernst}$ represents the reversible cell voltage, also known as the Nernst voltage, which can be calculated using Eq. (2)^67,68^.

2 $$V_{Nernst} = 1.229 - 0.85 \times 10^{ - 3} \left( {T_{stack} - 298.15} \right) + 4.3085 \times 10^{ - 5} T_{stack} \left[ {ln\left( {p_{{H_{2} }} } \right) + 0.5ln\left( {p_{{O_{2} }} } \right)} \right]$$

In this context, ${T}_{stack}$​ refers to the stack temperature measured in Kelvin (K), ${p}_{{H}_{2}}$ indicates the partial pressure of hydrogen in bars, and ${p}_{{O}_{2}}$ represents the partial pressure of oxygen, also measured in bars. The partial pressure of hydrogen is determined using Eq. (3).^66^.

3 $$p_{{H_{2} }} = 0.5 \cdot RH_{a} \cdot P_{{H_{2} O}}^{sat} \left[ {\left( {\exp \left( {\frac{{1.635\left( {\frac{{I_{fc} }}{{A_{cell} }}} \right)}}{{T_{stack}^{1.334} }}} \right) \times \frac{{RH_{a} \cdot P_{{H_{2} O}}^{sat} }}{{P_{a} }}} \right)^{ - 1} - 1} \right]$$

Calculating the partial pressure of oxygen at the cathode can be achieved by injecting pure oxygen into the FC’s cathode side according to Eq. (4).

4 $$p_{{O_{2} }} = P_{c} - \left( {RH_{c} \cdot P_{{H_{2} O}}^{sat} } \right) \cdot \left[ {\left( {\exp \left( {\frac{{4.192\left( {\frac{{I_{fc} }}{{A_{cell} }}} \right)}}{{T_{stack}^{1.334} }}} \right) \cdot \frac{{\left( {RH_{c} \cdot P_{{H_{2} O}}^{sat} } \right)}}{{P_{c} }}} \right)^{ - 1} - 1} \right]$$

If air replaces oxygen, the partial pressure of oxygen at the cathode may be computed using Eq. (5).

5 $$p_{{O_{2} }} = P_{c} - \left( {RH_{c} \cdot P_{{H_{2} O}}^{sat} } \right) - \frac{0.79}{{0.21}} \cdot p_{{O_{2} }} \cdot {\text{exp}}\left( {\frac{{0.291\left( {\frac{{I_{fc} }}{{A_{cell} }}} \right)}}{{T_{stack}^{0.832} }}} \right)$$

where $R{H}_{a}$ and $R{H}_{c}$ are the relative humidity of the vapours in the anode and cathode respectively. ${I}_{fc}$ is the current of operation of FC (A), ${A}_{cell}$ refers to the active cell area (cm2), ${P}_{a}$ represents pressure at anode (bar) while ${P}_{c}$ stands for pressure at cathode(bar). ${P}_{{H}_{2}O}^{sat}$ represent saturation pressure for water vapor (bar) which can be calculated as a function of stack temperature using Eq. (6).^67,68^.

6 $${\text{log}}_{10} \left( {P_{{H_{2} O}}^{sat} } \right) = 2.95 \times 10^{ - 2} \left( {T_{stack} - 273.15} \right) - 9.18 \times 10^{ - 5} \left( {T_{stack} - 273.15} \right)^{2} + 1.44 \times 10^{ - 7} \left( {T_{stack} - 273.15} \right)^{3} - 2.18$$

The Eq. given by (7)^67^ tells us that the activation polarization can be determined with the help of stack temperature and oxygen concentration.

7 $$V_{act} = - \left[ {\xi_{1} + \xi_{2} \cdot T_{stack} + \xi_{3} \cdot T_{stack} {\text{ln}}\left( {C_{{O_{2} }} } \right) + \xi_{4} \cdot T_{stack} \cdot {\text{ln}}\left( {I_{FC} } \right)} \right]$$

where ${\xi }_{k}(k=\text{1,2},\text{3,4})$ are coefficients of a semi-empirical equation derived from kinetic, thermodynamic and electrochemical theories^69^ and ${C}_{{O}_{2}}$ is the concentration of oxygen (mol ·cm − 3) that can be calculated by this equation^67^.

8 $$C_{{O_{2} }} = \left( {\frac{{p_{{O_{2} }} }}{5.08}} \right) \times 10^{6} {\text{exp}}\left( { - \frac{498}{{T_{stack} }}} \right)$$

As expressed in Eq. (9),^67^ the ohmic polarization relies on contact resistance, ${R}_{C}$ (Ω), and membrane resistance, ${R}_{m}$ (Ω).

9 $$V_{ohmic} = I_{FC} \cdot \left( {R_{m} + R_{C} } \right)$$

The membrane resistance depends on the resistivity of the membrane, ${\rho }_{m}$ (Ω.cm), membrane thickness, $l$ (cm), and effective membrane area (cm^2^), which is shown in Eq. (10).

10 $$R_{m} = \frac{{\rho_{m} l}}{{A_{cell} }}$$

The membrane resistivity (${\rho }_{m}$) is calculated by using Eq. (11) for Nafion membranes.

11 $$\rho_{m} = \frac{{181.6\left[ {1 + 0.03\left( {\frac{{I_{fc} }}{{A_{cell} }}} \right) + 0.062\left( {\frac{{T_{stack} }}{303}} \right)^{2} \left( J \right)^{2.5} } \right]}}{{\left[ {\lambda - 0.643 - 3\left( {\frac{{I_{fc} }}{{A_{cell} }}} \right)} \right]\exp \left( {4.18\left( {\frac{{T_{stack} - 303}}{{T_{stack} }}} \right)} \right)}}$$

where $\lambda$ is an adjustable parameter related to the membrane and its preparation process^69^. The concentration polarization is calculated using Eq. (12) ^67^.

12 $$V_{con} = - \beta {\text{ln}}\left( {1 - \frac{J}{{J_{max} }}} \right)$$

where $\beta$ is the parametric coefficient (V) that depends on the cell and its operation state^67^, $J$ is the actual current density (A $\cdot$ cm^−2^), and ${J}_{max}$ is the maximum current density (A $\cdot$ cm^−2^).

### Fitness function definition

In this research, the parameters of the model are optimized using various versions of Particle Swarm Optimization (PSO) and Finite Difference Differential Evolution (FD-DE) to align the PEMFC model’s results with those found in the literature or provided by manufacturers, thereby improving the model. The output voltage is calculated at points corresponding to each current value using the mathematical formulas detailed in the section titled "Semi-empirical Electrochemical Model." Consequently, the proposed fitness function acts as an indicator of the quality of the estimated parameters. The Sum of Squared Errors (SSE), presented in Eq. (13), is chosen as the fitness function^67^.

13 $$SSE = Min\left( {\mathop \sum \limits_{i = 1}^{N} \left[ {V_{meas} \left( i \right) - V_{calc} \left( i \right)} \right]^{2} } \right)$$

In this context, $N$ represents the total number of measured data points, $i$ is the iteration counter, ${V}_{meas}$ is the measured voltage of the Fuel Cell (FC), and ${V}_{calc}$​ refers to the voltage calculated for the FC. Additionally, various Multi-Attribute Decision Making (MADM) methods with differing foundational principles were outlined in the section "Ranking of the Algorithms." These methods are employed to determine the most effective Meta-Heuristic Algorithms (MHAs) for the H-1000 XP case study. The Mean Biased Error (MBE) is computed using Eq. (14).

14 $$MBE = \frac{{\mathop \sum \nolimits_{i = 1}^{N} \left| {V_{meas} \left( i \right) - V_{calc} \left( i \right)} \right|}}{N}$$

## Enhanced artificial hummingbird algorithm

### Principles of the artificial hummingbird algorithm

The first step of the Artificial Hummingbird Algorithm (AHA) is to generate candidate solutions that are distributed randomly. When an artificial hummingbird comes to a newer area, it will randomly find food sources in order to complete the establishment of colony. This behavior represents the first search for food sources. The process of initialization is given by Eq. (15):

15 $$x_{i} = LB + r \cdot \left( {UB - LB} \right)$$

Here, LB and UB represent the upper and lower bounds of the interval, respectively, while $r$ is a random number between 0 and 1. The variable ${x}_{i}$​ denotes the position identified by the $i$ th hummingbird.

The visit table is put like this:

16 $$VT_{i,j} = \left\{ {\begin{array}{*{20}c} {0,} & {if\;i \ne j} \\ {null,} & {i = j} \\ \end{array} } \right.$$

For the first scenario, ${\text{VT}}_{ij}$=null indicates that hummingbirds are feeding at a consistent food source and for the second scenario, ${\text{VT}}_{ij}$=0 indicates that the $i$th hummingbird has recently explored the $j$th food source.

Three flight trajectories are used by hummingbirds for multidimensional space navigation. The axial flight is important because it allows the bird to move along the axis, as explained in Eq. (17).

17 $$D_{Af} ^{\left( i \right)} = \left\{ {\begin{array}{*{20}l} {1,} \hfill & {{\text{if}}\;{ }i = {\text{Randi}}\left( {\left[ {1,d} \right]} \right)} \hfill \\ {0,} \hfill & {\text{else }} \hfill \\ \end{array} } \right.$$

The diagonal flight can be expressed by Eq. (18).

18 $$D_{Df} ^{\left( i \right)} = \left\{ {\begin{array}{*{20}l} {1,} \hfill & {{\text{if}}\;i = G\left( j \right),\;j \in \left[ {1,c} \right],\;G = {\text{ Randperm }}\left( c \right)} \hfill \\ {0,} \hfill & {{\text{else }},} \hfill \\ \end{array} } \right.$$

The omnidirectional flight is expressed by Eq. (19).

19 $$D_{Of}^{\left( i \right)} = 1,{ }i = 1,2, \ldots ,n$$

In this model, $c$ takes a number of values between 2 and $\left[{r}_{1}(d-2)+1\right],$ where $\text{Randi}([1,d])$ generates a random number between 1 and $d$, whereas $Randperm (c)$ generates an arrangement of numbers randomly chosen from the set of all whole numbers smaller than $c$. ${r}_{1}$ is a randomly generated quantity that comes from within the interval (0,1).

The equation of the hummingbird’s candidate solutions update during guided foraging is illustrated in Eq. 20.

20 $$v_{i} \left( {t + 1} \right) = x_{{i,\;{\text{target}}}} + g \cdot D_{t} \cdot \left( {x_{i} \left( t \right) - x_{{i,\;{\text{target }}}} \left( t \right)} \right),\quad t \in \left\{ {Af,Df,Of} \right\}$$

21 $$g \sim N\left( {0,1} \right)$$

The position of the target solution is represented by ${x}_{i,\text{target}}(t)$ and $g$ is a factor that guides it. The update formula, which applies when a hummingbird finds the target food source closer to its location, is given below:

22 $$x_{i} \left( {t + 1} \right) = \left\{ {\begin{array}{*{20}l} {x_{i} \left( t \right),} \hfill & {f\left( {x_{i} \left( t \right)} \right) \le f\left( {v_{i} \left( {t + 1} \right)} \right)} \hfill \\ {v_{i} \left( {t + 1} \right),} \hfill & {\text{else }} \hfill \\ \end{array} } \right.$$

The fitness values of the candidate solution ${x}_{i}(t)$ and the updated solution ${v}_{i}(t+1)$ are represented by $f\left({x}_{i}(t)\right)$ and $f\left({v}_{i}(t+1)\right)$ respectively.

The equation for revising candidate solutions through territorial foraging by hummingbirds is given in Eq. (23).

23 $$v_{i} \left( {t + 1} \right) = x_{i} + k \cdot D_{t} \cdot x_{i} \left( t \right),\quad t \in \left\{ {Af,Df,Of} \right\}$$

24 $$k \sim N\left( {0,1} \right)$$

$k$ is a guiding parameter, and ${D}_{t}$ stands for one of the three modes of flight. The following outlines the strategy for insufficiently placed artificial hummingbirds migrating to another food source by way of migratory foraging.

25 $$x_{{\text{worst }}} \left( {t + 1} \right) = LB + r \cdot \left( {UB - LB} \right),\;{\text{when}}\;M = t$$

where ${x}_{\text{worst}}$ shows the candidate solution with lowest nectar refilling rate, $t$ is the current iteration number and $M$ denotes migration coefficient used in the proposed algorithm. Normally the value of $M$ is set as $M=2n$ where $n$ represents population size.

### LCAHA algorithm: framework

The Artificial Hummingbird Algorithm (AHA), which requires an improvement to handle engineering optimization problems that possess multiple local optimal solutions, is enhanced by means of a more advanced hybrid version called the multi-strategy hybrid AHA. In this version, sinusoidal chaotic maps, Lévy flight and cross-and-update foraging strategy have been integrated.

#### Sinusoidal chaotic map strategy

The unpredictable, unsteady and undetermined nature of such maps as chaotic mappings in nonlinear dynamics is well-known^70^. Chaotic variables are used to start the population of these maps and hence offer a more exhaustive searching process than random searches which heavily depend on probabilities^71^. Moreover, chaotic maps are made effective by their sensitivity to initial conditions and parameters^72^. This research utilizes one-dimensional mapping that is done using the sinusoidal chaotic map, which creates a wider exploration through iterative initialization process into the search space.

26 $$\left\{ {\begin{array}{*{20}l} {f_{j + 1} = \alpha f_{j}^{2} {\text{sin}}\left( {\pi f_{j} } \right)} \hfill \\ {f_{0} \in \left[ {0,1} \right],\alpha \in \left( {0,4} \right]} \hfill \\ \end{array} } \right.$$

where $j$ represents the quantity of iterations. The formula increases the range of search through initiating process of iterations to understand what happens where.

#### The Lévy flight introduction

Lévy flight is a frequent behavior exhibited by many flying animals and is comprised of random walks with a heavy-tailed probability density function that encompasses lots of small steps as well as rare long jumps^73–75^. For populations that generally move toward predetermined food sources but have to search for new prey sites, this type of movement is very efficient^76^. As such, Lévy flight has extensively enhanced the efficiency of several Swarm Intelligence (SI) algorithms^77^.

By incorporating Lévy flight, the integration of guided foraging allows artificial hummingbirds to efficiently determine the precise area where target food sources are located, expand their survey areas, in surrounding regions and improve the diversity of search process as a whole. Guided foraging which includes Lévy flight is defined by Eq. (27).

27 $$v_{i} \left( {t + 1} \right) = x_{{i,\;{\text{target }}}} + \alpha \cdot {\text{ Levy}} \cdot D_{t} \cdot \left( {x_{i} \left( t \right) - x_{{i,\;{\text{target }}}} \left( t \right)} \right){, }\quad t \in \left\{ {Af,Df,Of} \right\}$$

where $\alpha$ is set to 0.01.

28 $${\text{Levy}}\left( \beta \right) \sim u = t^{ - 1 - \beta } ,\;0 < \beta \le 2$$

Also, Lévy flight’s effect is influenced by two factors: the impact of uniform distribution on the flight direction and the influence of Lévy distribution on step length.

29 $$s = \frac{U}{{|V|^{1/\beta } }}$$

where $U$ and $V$ obey Gaussian distribution as illustrated in Eq. (30).

30 $$U \sim N\left( {0,\sigma_{U}^{2} } \right),\;V \sim N\left( {0,\sigma_{V}^{2} } \right)$$

where ${\sigma }_{U}$ and ${\sigma }_{V}$ satisfy Eqs. (31) and (32):

31 $$\sigma_{U} = \left( {\frac{{{\Gamma }\left( {1 + \beta } \right) \cdot {\text{sin}}\left( {\pi \cdot \beta /2} \right)}}{{{\Gamma }\left( {\left( {1 + \beta /2} \right) \cdot \beta \cdot 2^{{\left( {\beta - 1} \right)/2}} } \right)}}} \right)^{1/\beta }$$

32 $$\sigma_{V} = 1$$

where $\Gamma$ represents the standard Gamma function, and β\betaβ is set at 1.5^78^.

#### Cross and update foraging strategy

Crossover and update foraging strategies used in this study are conducted following the crossover operator from CSO. The operator employs previous iterations’ information to increase future search ability^79^. The crossover operator is an improved catalyst that effectively identifies the best quality solutions. It is divided into two parts, namely; horizontal and vertical operators^80^. These operators shift the population iteratively until they reach the optimum position. The Cross foraging simulates artificial hummingbirds exchanging positional information while Update foraging is how hummingbirds change their information processing due to a changing environment with which they interact during their lifetime in search of nectar. In this case, these strategies can reproduce both fundamental communication behaviors between artificial hummingbirds and environmental characteristics of nectar sources. During evolutionary iterations, potential new food locations found by hummingbird using movements across horizontal and vertical directions are considered as candidate honey sources.

##### Horizontal operator-based cross foraging strategy

Information trading among artificial hummingbirds is the main issue of this study. The horizontal operator acts as a bridge to span across the solution space and exchange some information. It’s called “cross foraging” because of the horizontal operator that discovered how population level information transfer can be efficient. This is why sharing the current feeding sources by two types of artificial hummingbirds enables them to know where a new source of honey may be found for influencing their positional updates. Hence, with this strategy, both birds can avoid local maxima by searching broadly. Therefore, we can characterize the updating mechanism for cross foraging as:

33 $$V_{hc} \left( {N_{1} ,j} \right) = r_{1} \cdot x\left( {N_{1} ,j} \right) + \left( {1 - r_{1} } \right) \cdot x\left( {N_{2} ,j} \right) + c_{1} \cdot \left( {x\left( {N_{1} ,j} \right) - x\left( {N_{2} ,j} \right)} \right),$$

34 $$V_{hc} \left( {N_{2} ,j} \right) = r_{2} \cdot x\left( {N_{2} ,j} \right) + \left( {1 - r_{2} } \right) \cdot x\left( {N_{1} ,j} \right) + c_{1} \cdot \left( {x\left( {N_{1} ,j} \right) - x\left( {N_{2} ,j} \right)} \right)$$

Two food sources $x\left({N}_{1},j\right)$ and $x\left({N}_{2},j\right)$ are presented after one iteration of applying horizontal operator in the $dth$ dimension. In this case, ${r}_{1}$ and ${r}_{2}$ are random numbers between (0,1). Besides, ${c}_{1}$ and ${c}_{2}$ are stochastic coefficients for [-1, 1] and ${V}_{hc}\left({N}_{1},d\right)$ as well as ${V}_{hc}\left({N}_{2},d\right)$ are brand-new candidate honey sources because of information sharing between two hummingbirds.

With the help of expansion factors ${c}_{1}$ and ${c}_{2}$, a horizontal operator can find new positions at the hypercube edges with certain probability. This way, the LCAHA reduces its blind spots which could hinder hummingbirds from finding food sources. It greatly improves their global search ability.

##### Revised foraging strategy utilizing the vertical operator

To continue investigating the effects of changes in environmental information on flight behavior, a revised foraging strategy has been developed that includes a vertical operator mechanism. The flight of these man-made creatures is affected by the change in environmental conditions such as temperature, illumination and humidity when they are heading towards probable sources of nectar. These environmental factors alter their certainty towards these sources hence can change their path to be taken. As a result of this behavior, they might end up in unexplored regions where there is possibility of discovering new nectar sources. Artificial hummingbirds are able to enter previously unknown territories through an adaptive strategy which enhances global exploration and local exploitation capabilities. As well, it addresses problems connected with recurred stops during multiple iterations. This approach uniquely modifies the positions of nectar sources by applying two dimensional planes across different locations where there is food for them using vertical operations.

The revised strategy utilizes vertical manipulations in the dimensions ${d}_{1}$​ and ${d}_{2}$​ of an artificial hummingbird’s position $x(i,:)$, updating the position of a new potential nectar source ${V}_{vc}(i)$ as outlined below:

35 $$V_{vc} \left( {i,d_{1} } \right) = c \cdot x_{{i,d_{1} }} + \left( {1 - c} \right) \cdot x_{{i,d_{2} }} ,{ }i \in M\left( {1,n} \right),{ }d_{1} ,d_{2} \in M\left( {1,{\text{Dim}}} \right)$$

where $c$ represents a random number within the range $(\text{0,1})$. Here, $n$ signifies the agents, and $Dim$ refers to the design variables.

In this context, the population is normalized based on the upper and lower bounds of each design variable. Each vertical operation is dedicated to a single nectar source, avoiding the risk of disrupting another potentially optimal global dimension by exiting a locally optimal stale dimension.

Additionally, a competitive operator is introduced to manage the nectar-refilling dynamics between the new and existing nectar sources. A newly discovered nectar source by an artificial hummingbird is not immediately adopted; it is only considered if its nectar-refilling rate surpasses that of the current source. The mathematical expression for this competitive operator is given as:

36 $$x = \left\{ {\begin{array}{*{20}l} {V_{hv} ,} \hfill & {{\text{if }}\;f\left( {V_{hv} } \right) < f\left( x \right),} \hfill \\ {x,} \hfill & {{\text{if}}\;{ }f\left( {V_{hv} } \right) > f\left( x \right),} \hfill \\ \end{array} } \right.$$

where ${V}_{hv}$ is the candidate honey source obtained after competitive arithmetic.

### Equilibrium between exploration and exploitation

Exploration and exploitation together form a comprehensive search strategy. The exploration mechanism extends the search by identifying and pushing candidate solutions towards unexplored areas far within the search space. Conversely, exploitation drives the solutions to converge towards the most promising regions identified. A careful equilibrium between these two opposing functions directs the algorithm towards optimal performance.

Initially, the inclusion of Lévy flight macro migration introduces frequent minor movements that diversify the motion of search agents. This strategy, adopted during the exploratory phase, allows candidate solutions to bypass local optima via the Lévy step size, enhancing the overall search effectiveness globally. This approach not only stabilizes the global search but also maintains a balance between exploration and exploitation. Moreover, the implementation of a horizontal operator within the cross-foraging strategy incorporates an expansion factor ${c}_{1}$​, enabling the sampling of new positions at the hypercube’s edges with minimal probability. This tactic minimizes unsearchable blind spots by the primary agent, thereby boosting the global search capabilities of the Artificial Hummingbird Algorithm (AHA). Furthermore, the adoption of vertical operators ensures normalization of the hummingbird population based on each dimension’s upper and lower limits. Simultaneously, each vertical crossover operation produces a single new candidate solution, offering a chance for the search to escape local optima in stagnant dimensions without negatively impacting other dimensions that might represent the global optimum. The dual-stage enhancement effectively balances exploration and exploitation.

### Optimization process steps for the LCAHA

In order to solve complex, high-dimensional engineering problems, the Artificial Hummingbird Algorithm (AHA) should tackle challenges such as local optima, slow convergence and limited exploration capabilities. A multi-strategy improved version of the AHA was developed for this purpose known as Hybrid Artificial Hummingbird Algorithm (LCAHA). This new approach includes sinusoidal chaotic maps, Lévy flight and advanced cross and update foraging strategies which have a number of advantages:

1. Improved Solution Distribution: Within the initial distribution in solution space sinusoidal chaos maps are integrated to cover more search area and lead to faster convergence towards optimum solution. Thus LCAHA has an increased convergence speed with higher accuracy.
2. Greater Population Diversity: The inclusion of Levy flight increases diversity among artificial hummingbird populations eliminating their premature convergence. As a result, rather than getting stuck on some suboptimal solutions LCAHA better escapes from local optima hence achieving more efficiency when it comes to identifying global optimal solutions at different stages of optimization process.
3. Better Exploration and Exploitation: The algorithm is improved by using cross and update foraging strategies that provide updated information about where the birds are located at both population level and dimension level resulting into balance between exploration and exploitation processes which enable detailed searching optimal solutions within the solution space.

The procedural steps for implementing LCAHA are as follows:

- **Step 1:** Set initial LCAHA parameters: number of agents $n$, design variables $Dim$, boundaries of variables $(lb,ub)$, maximum iterations $Max\_Iteration$, and migration coefficient $M$.
- **Step 2:** Randomly initialize $n$ food sources using sinusoidal chaotic maps and set up the initial visit table per Eq. (16).
- **Step 3:** Hummingbirds approach the nearest food source, assessing and recording the highest nectar-refilling rate and optimal food source $best (x);$
- **Step 4:** During each iteration, generate a random number ${r}_{1}$​ within [0,1]. Based on ${r}_{1}$​, hummingbirds employ axial, diagonal, or omnidirectional flights as prescribed by Eqs. (17), (18), or (19), respectively.
- **Step 5:** Generate another random $r$ within [0,1]. If $r\le 0.5$, hummingbirds engage in guided foraging via Eq. (27) using Lévy flight to assess nearby food sources.
- **Step 6:** If $r>0.5$, adjust the hummingbird’s location through territorial foraging as defined in Eq. (23), and assess the nectar-refilling rate of the new source. If it proves better, switch to the new optimal solution $best\left(x\right)$ and reset the food source record.
- **Step 7:** Every $2n$ iterations, update the position by migratory foraging using Eq. (25), relocating the least efficient hummingbird to a new food source.
- **Step 8:** Update positions and explore new food sources using Eqs. (35) and (36) through the cross and update foraging strategy, evaluating the nectar-refilling rate for potential updates.
- **Step 9:** After each iteration, increase $t$; if $t$ exceeds $Max\_Iteration$, declare the global minimum and optimum variables; if not, return to Step 4.

To distinctly outline the multi-strategy hybrid AHA, Algorithm 1 provides the pseudo-code for the advanced LCAHA.

**Algorithm 1:**
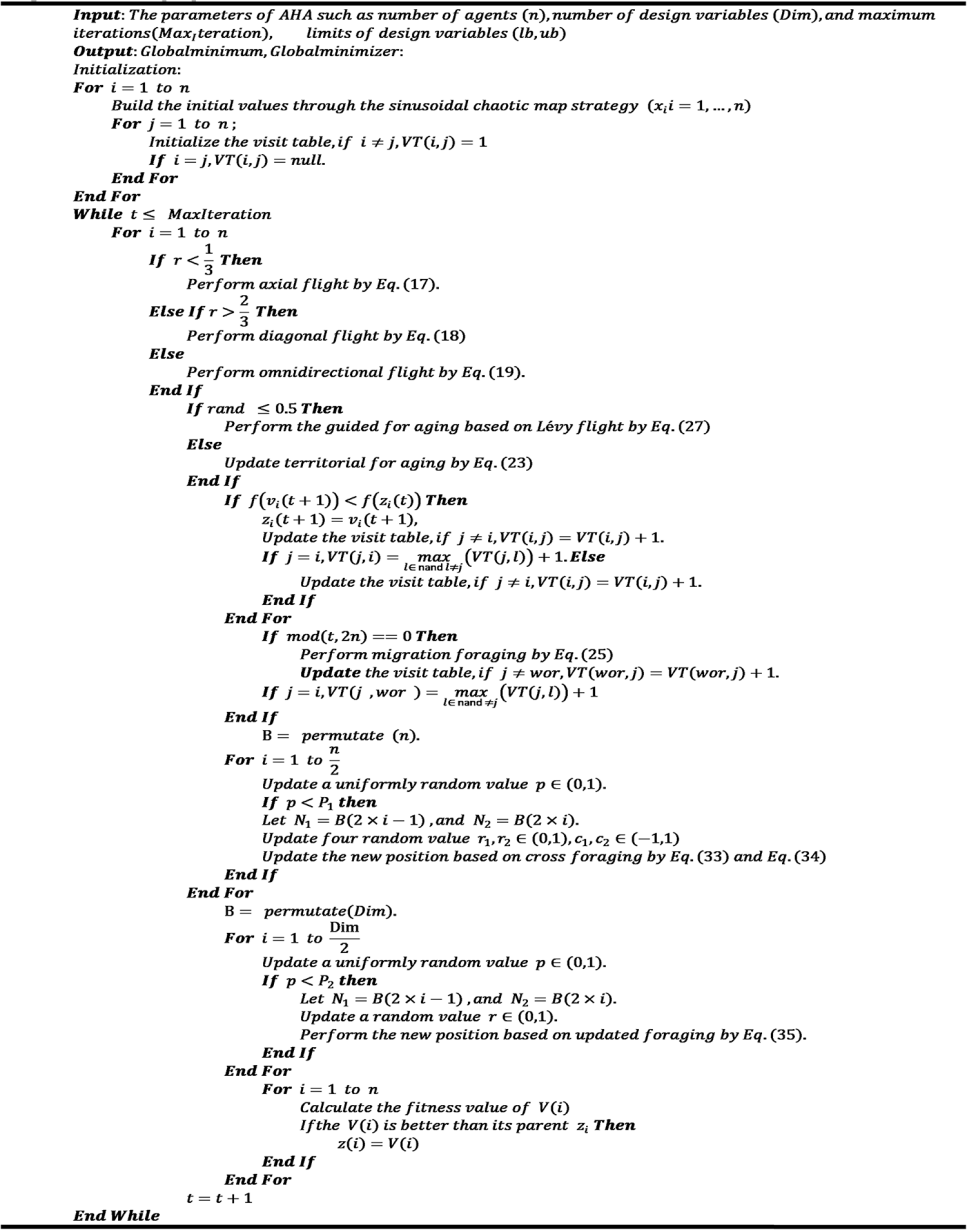
The proposed LCAHA

**Fig. 1:**
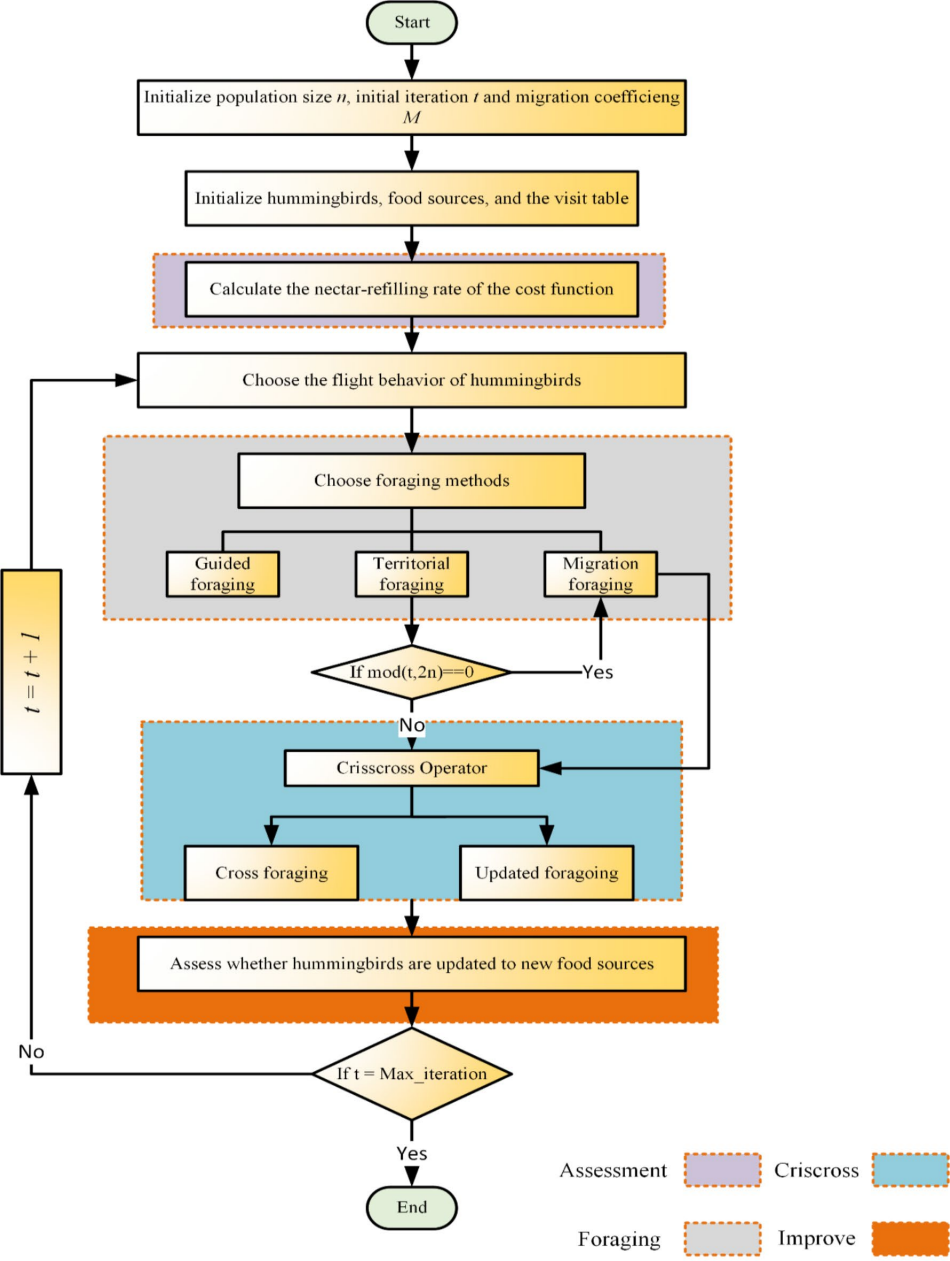
Flowchart for the LCAHA.

Moreover, Fig. 1 depicts the flowchart of the LCAHA algorithm with emphasis on three essential improvement approaches. To begin with, algorithm parameters have been set up and initial evaluation has been done. Then, both global and local searches are performed by LCAHA. The iterative outcomes are refined through cross and update foraging strategy until the termination criteria is met which concludes the process. Eventually, in output mode, algorithm produces optimally improved solution. The use of parameters of chaotic mapping, Lévy flight, and the cross and update foraging strategy, where parameters such as the migration coefficient (M), guiding factors (α, g, k) and the sinusoidal chaotic map constant α (0–4) are set to guarantee efficient optimization. The contribution of these parameters is to improve the balance between exploration and exploitation of the LCAHA for PEMFC parameter estimation, which leads to the improvement of the convergence rate and accuracy.

### Computational complexity of LCAHA

Five main aspects and among them are the initial phase, number of hummingbirds ($n$), Max_Iteration ($M$) and the number of design variables ($d$) determine time complexity of LCAHA algorithm. At the same time, before any iterations, metaheuristic algorithm initializes total dimensionality for all individuals in the population. The cost to initialize LCAHA is $O(n \times d)$. Since an objective function’s form may depend on a problem type and can’t be standardized, its time complexity may not be a significant factor considered about it. According to the AHA introduction, at each iteration guided foraging or territorial foraging done with 50% chance have computational complexities of $O(0.5M\times n\times d) and O(0.5M\times n\times d)$ respectively due to their position updates while migratory foraging only occurs in half iterations hence having complexity $O(M\times d/2)$. Cross-foraging on the other hand has a complexity of $O(M\times n\times d)$ since it swaps positions between pairs of individuals in population simultaneously. Changing from one foraging strategy into another takes place by updating only some dimensions thereby resulting in complexity $O(M\times n)$, where only one update happens. Thus, overall this can be written as:

The computational complexity of LCAHA primarily hinges on five factors: the initialization phase, the number of hummingbirds $(n)$, maximum iterations $(M)$, and the number of design variables $(d)$. The initialization of the metaheuristic algorithm, which sets up the total dimensions for all individuals before beginning the iterations, has a complexity of $O(n \times d)$. The complexity related to solving the objective function is not included here, as it varies depending on the problem type and cannot be universally applied. As detailed in AHA, guided and territorial foraging occurs during each iteration with equal probability, attributing a computational complexity of $O(0.5M\times n\times d)$ and $O(0.5M\times n\times d)$ for each. Furthermore, migratory foraging, conducted in half of the iterations, contributes a complexity of $O(M\times d/2)$. Cross foraging, which involves positional exchanges between pairs of individuals, also adds a complexity of $O(M\times n\times d)$. Update foraging, which adjusts specific dimensions, brings the complexity to O(M $\times$ n). Thus, the overall complexity of LCAHA can be summarized as follows:

$$\begin{aligned} & O\left( {LCAHA} \right) = O\left( {Initializationphase} \right) + O\left( {objectivefunction} \right) + O\left( {guidedforaging} \right) \\ & \quad \quad + O\left( {territorialforaging} \right) + O\left( {migratoryforaging} \right) + O\left( {crossforaging} \right) \\ & \quad \quad + O\left( {updateforaging} \right) \\ & \quad = O\left( {n \times d} \right) + O\left( {0.5M \times n \times d} \right) + O\left( {0.5M \times n \times d} \right) + O\left( {M \times d/2} \right) + O\left( {M \times n \times d} \right) + O\left( {M \times n} \right) \\ & \quad = O\left( {n \times d + 2M \times n \times d + M \times d/2 + M \times n} \right) \approx O\left( {2M \times n \times d + M \times d/2 + M \times n} \right). \\ \end{aligned}$$

## Result analysis and discussion

In this work an attempt has been made to exhaustively illustrate LCAHA algorithm and compare it with different highly applied algorithms like Particle Swarm Optimization (PSO)^58^, Differential Evaluation (DE)^59^, Grey Wolf Optimizer (GWO)^60^ and Sparrow Search Algorithm (SSA)^61^, applied for PEMFC modelling. The default parameter settings for different algorithms used in literatures are given in Table 1. All algorithms compared were set to their recommended to estimate the parameter of a PEMFC fuel cell (BCS 500W-PEM^62^, 500W-SR-12PEM^63^, Nedstack PS6^63^, 12 W-HR-12 PEM^64^, 500WHORIZON PEM^64^ and 250W-stack^65^) presented in Table 2**.** All the experiments are carried out on Matlab 2021a of a PC with Windows Server 2019 operating system CPU i7-11700 k@3.6 GHz, maximum iterations 500, number of run 50 and population size 40.

**Table 1:** Default parameter settings of the compared algorithms.

Algorithms	Default settings
PSO	Inertia weight = Linear decrease from 0.9 to 0.1; Velocity range = 0.1 times the variable range, Cognitive and social factors ${c}_{1}=2; {c}_{2}=2$
DE	Scaling factor = 0.5, Crossover probability = 0.5
GWO	Convergence parameter (a) Linear reduction from 2 to 0
SSA	*ST* = *0.8*
LCAHA	$\alpha =0.01$

**Table 2:** Characteristics of Six PEMFCs used in this work.

S. no	PEMFC type	Power(W)	Ncells (no)	A(cm^2^)	l(um)	T(K)	Jmax(mA/cm^2^)	PH_2_(bar)	PO_2_(bar)
FC1	BCS 500 W	500	32	64	178	333	469	1.0	0.2095
FC2	NetStack PS6	6000	65	240	178	343	1125	1.0	1.0
FC3	SR-12	500	48	62.5	25	323	672	1.47628	0.2095
FC4	H-12	12	13	8.1	25	323	246.9	0.4935	1.0
FC5	STD	250	24	27	127	343	860	1.0	1.0
FC6	Horizon	500	36	52	25	338	446	0.55	1.0

### FC1: BCS 500W

**Fig. 2:**
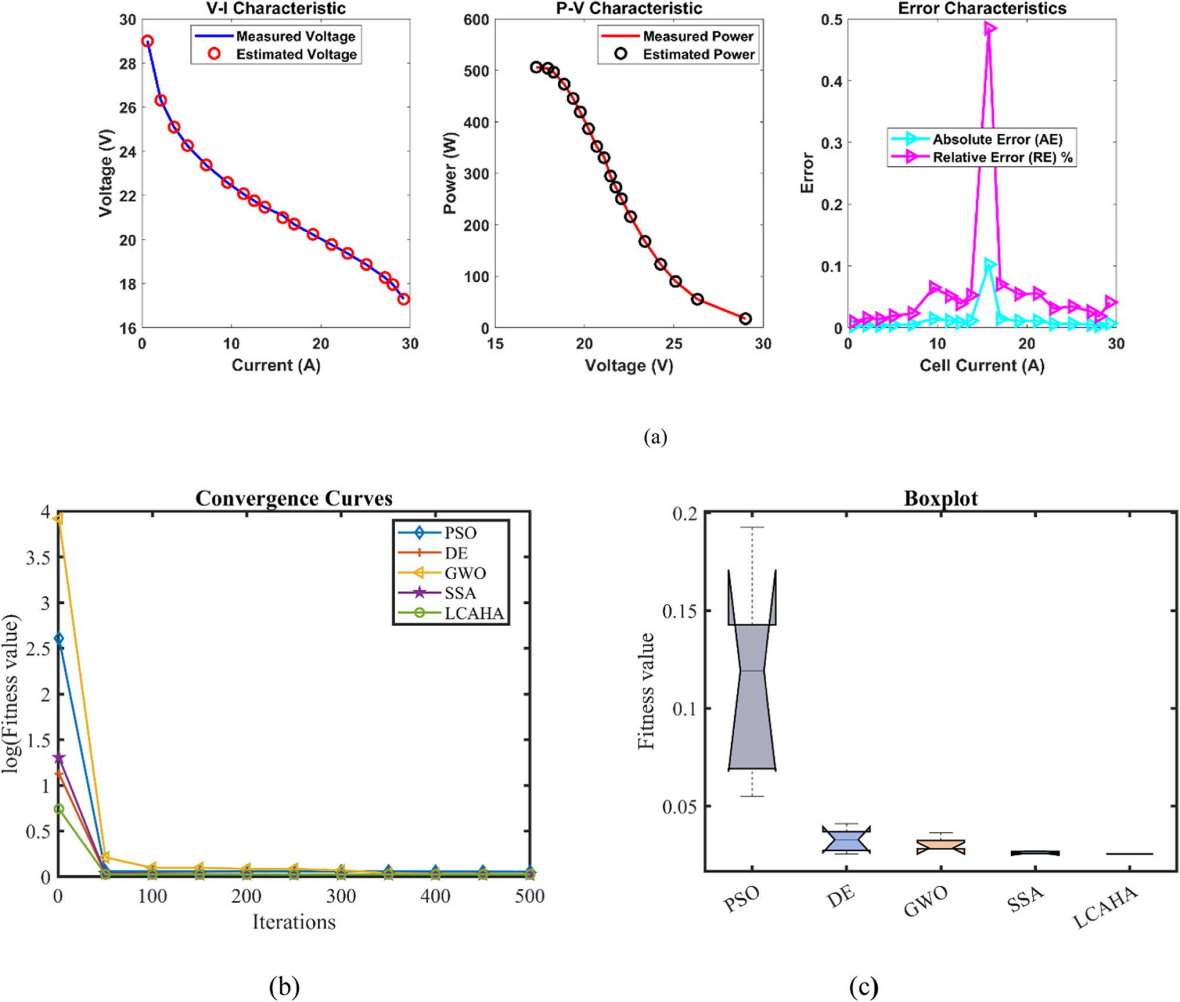
FC1 (**a**) V-I, P–V and Error Curve, (**b**) Convergence Curve, (**c**) Box-Plot.

According to Table 3, the LCAHA algorithm consistently delivers either the lowest or among the lowest values in all evaluated categories, showcasing its superior stability, precision, and effectiveness. The algorithm’s minimum value is recorded at 0.0254927, matching DE for the lowest among the compared algorithms, thereby illustrating its consistent ability to identify optimal solutions. The maximum value for LCAHA remains constant at 0.0254928, notably lower than those observed with PSO (0.1924899) and GWO (0.0364916), which highlights its capacity to avoid scenarios with high error rates. The mean value of LCAHA is also the most favorable at 0.0254927, confirming its ability to produce reliably accurate outcomes. Additionally, LCAHA exhibits remarkable stability, as evidenced by an extremely low standard deviation of 4.59E-08, much lower than those seen in PSO (0.053443) and DE (0.0061464), indicating its unparalleled precision. In terms of computational speed, LCAHA demonstrates impressive efficiency with a runtime of 2.8059648 s, which is faster than both DE, which takes 6.5825206 s, and SSA, which requires 5.9258096 s. Furthermore, LCAHA achieves the best Friedman rank (FR) at 1.2, solidifying its position as the most efficient algorithm across all considered measures. As evidenced by the data in Tables 3, 4, and Fig. 2, LCAHA not only excels in delivering top-tier results with minimal computational demand but also consistently outperforms other algorithms in terms of stability and efficiency, positioning it as the optimal choice for precision-critical and time-sensitive applications.

**Table 3:** Optimized parameters and optimal function value for FC1.

Algorithm	PSO	DE	GWO	SSA	LCAHA
${{\varvec{\xi}}}_{1}$	−0.9840126	−0.8721622	−1.1105925	−1.1504024	−0.9259692
${{\varvec{\xi}}}_{2}$	0.00301	0.0022555	0.0031004	0.0036846	0.0026293
${{\varvec{\xi}}}_{3}$	6.454E−05	3.718E−05	4.552E−05	7.538E−05	5.084E−05
${{\varvec{\xi}}}_{4}$	−0.0001814	−0.000193	−0.0001908	−0.0001928	−0.000193
${\varvec{\lambda}}$	20.681348	20.877275	21.252609	22.094647	20.877243
${{\varvec{R}}}_{{\varvec{c}}}$	0.0007508	0.0001	0.0003099	0.0002184	0.0001
***B***	0.0136	0.0161261	0.0151479	0.0161511	0.0161261
***Min***	0.0550084	0.0254927	0.0280372	0.0256024	0.0254927
***Max***	0.1924899	0.0410172	0.0364916	0.0270263	0.0254928
***Mean***	0.1133755	0.0325788	0.0304626	0.0261257	0.0254927
***Std***	0.053443	0.0061464	0.0035903	0.0006616	4.587E−08
***RT***	3.7415366	6.5825206	2.8088295	5.9258096	2.8059648
***FR***	5	3	3.6	2.2	1.2

**Table 4:** Performance metrics of LCAHA Algorithm for FC1.

*S. NO*	*I*_*exp*_* (A)*	*V*_*exp*_* (V)*	*V*_*est*_* (V)*	*P*_*exp*_* (W)*	*P*_*est*_* (W)*	*AE*_*v*_* (A)*	*RE %*	*MBE*
1	0.6	29	28.997222	17.400000	17.398333	0.002778	0.009579	4.28697E−07
2	2.1	26.31	26.305940	55.251000	55.242475	0.004060	0.015430	9.15584E−07
3	3.58	25.09	25.093560	89.822200	89.834946	0.003560	0.014191	7.04273E−07
4	5.08	24.25	24.254627	123.190000	123.213504	0.004627	0.019079	1.18928E−06
5	7.17	23.37	23.375424	167.562900	167.601788	0.005424	0.023208	1.63429E−06
6	9.55	22.57	22.584624	215.543500	215.683157	0.014624	0.064793	1.18807E−05
7	11.35	22.06	22.071337	250.381000	250.509672	0.011337	0.051391	7.14011E−06
8	12.54	21.75	21.758473	272.745000	272.851254	0.008473	0.038957	3.98861E−06
9	13.73	21.45	21.461273	294.508500	294.663273	0.011273	0.052553	7.05953E−06
10	15.73	21.09	20.987752	331.745700	330.137340	0.102248	0.484817	0.000580813
11	17.02	20.68	20.694520	351.973600	352.220734	0.014520	0.070214	1.17132E−05
12	19.11	20.22	20.230997	386.404200	386.614357	0.010997	0.054388	6.71885E−06
13	21.2	19.76	19.770955	418.912000	419.144244	0.010955	0.055440	6.66721E−06
14	23	19.36	19.366037	445.280000	445.418843	0.006037	0.031181	2.0245E−06
15	25.08	18.86	18.866479	473.008800	473.171281	0.006479	0.034351	2.33173E−06
16	27.17	18.27	18.274733	496.395900	496.524497	0.004733	0.025906	1.24455E−06
17	28.06	17.95	17.953323	503.677000	503.770254	0.003323	0.018515	6.13598E−07
18	29.26	17.3	17.292890	506.198000	505.989949	0.007110	0.041101	2.80879E−06
Average value of different datasheets						0.012920	0.061394	3.61043E−05

### FC2: NetStack PS6

**Fig. 3:**
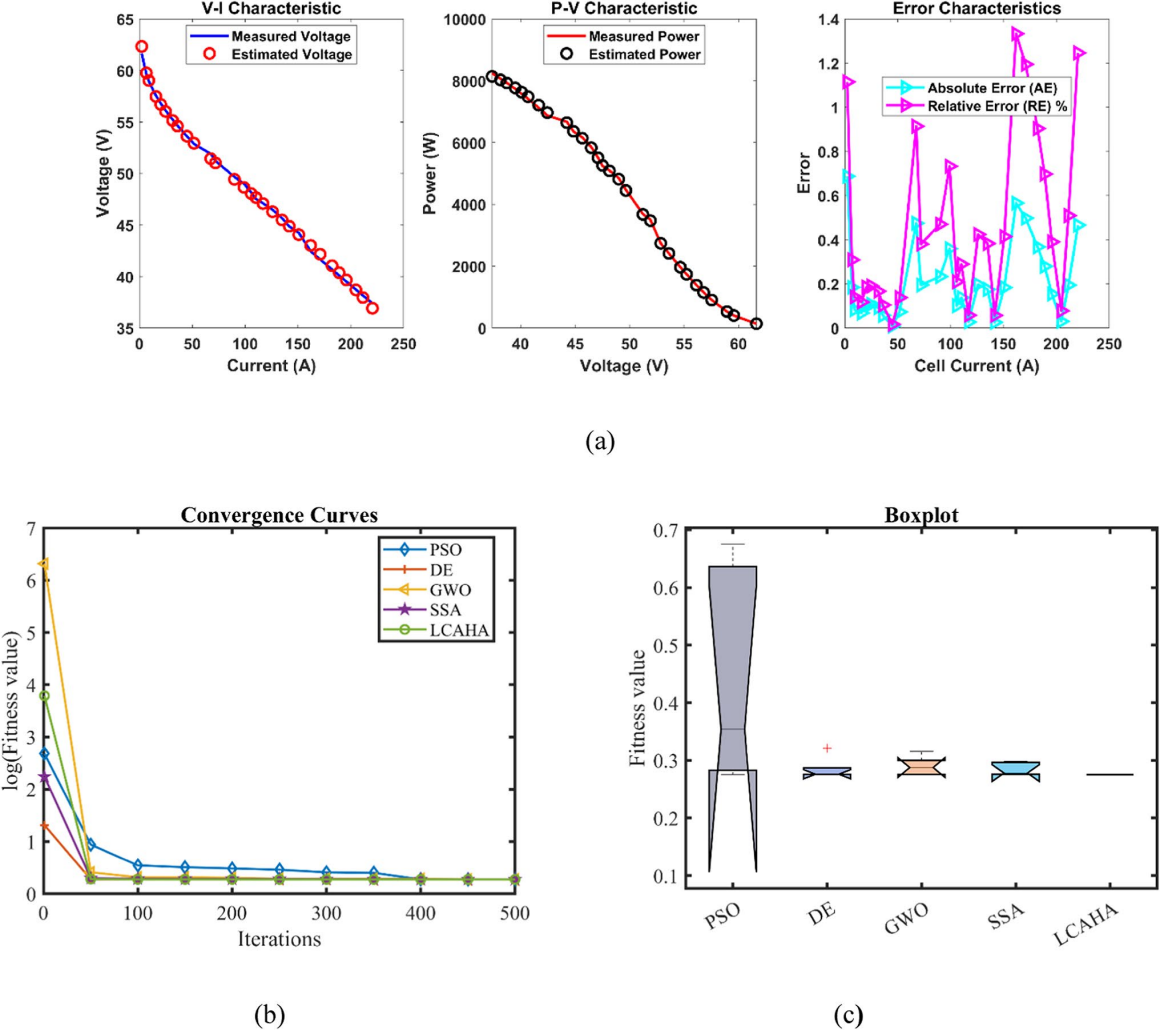
FC2 (**a**) V-I, P–V and Error Curve, (**b**) Convergence Curve, (**c**) Box-Plot.

Table 5 reveals that the LCAHA algorithm consistently records the lowest or nearly the lowest values, affirming its exceptional stability, precision, and effectiveness. LCAHA achieves a minimum value of 0.2752105, the best among all competing algorithms, illustrating its consistent capacity for identifying optimal solutions. Furthermore, this value also stands as the maximum, markedly better than PSO (0.6747868) and GWO (0.3155648), thus showcasing its strength in steering clear of high-error instances. The algorithm’s mean value, identical to its minimum and maximum, at 0.2752105, underscores its consistent accuracy in outcomes. LCAHA exhibits extraordinary stability, evidenced by an extremely low standard deviation of 2.51E-16, considerably lower than the variability seen in PSO (0.1917348) and DE (0.0199925), highlighting its superior precision. When it comes to computational speed, LCAHA shows great efficiency with a runtime of 3.9154243 s, outperforming GWO (9.410101 s) and SSA (8.2868594 s). Additionally, it achieves the highest Friedman rank (FR) of 1, further solidifying its status as the most effective algorithm across all evaluated measures. As detailed in Tables 5 and 6, and illustrated in Fig. 3, LCAHA not only excels in delivering outstanding results with minimal computational demands but also consistently surpasses other algorithms in terms of stability and efficiency. This makes it the premier choice for applications requiring high precision and time efficiency.

**Table 5:** Optimized parameters and optimal function value for FC2.

Algorithm	PSO	DE	GWO	SSA	LCAHA
${\xi }_{1}$	−1.1038486	−1.19969	−0.9284291	−0.8995119	−0.9841445
${\xi }_{2}$	0.0037071	0.0039192	0.0033298	0.002554	0.0028593
${\xi }_{3}$	7.716E−05	7.254E−05	8.689E−05	3.747E−05	4.165E−05
${\xi }_{4}$	−0.0000954	−0.0000954	−0.0000954	−9.542E−05	−0.0000954
$\lambda$	14	14	14	14.093636	14
${R}_{c}$	0.0001363	0.0001	0.0001063	0.0001195	0.0001204
*B*	0.0146781	0.019593	0.0187448	0.0180104	0.0167879
*Min*	0.2756414	0.2759	0.2755531	0.2759128	0.2752105
*Max*	0.6747868	0.3206847	0.3155648	0.2983612	0.2752105
*Mean*	0.4427205	0.2849213	0.2898211	0.2849642	0.2752105
*Std*	0.1917348	0.0199925	0.0165501	0.0112792	2.513E−16
*RT*	4.5417768	4.1026754	9.410101	8.2868594	3.9154243
*FR*	4.2	3	3.2	3.6	1

**Table 6:** Performance metrics of LCAHA Algorithm for FC2.

*S. NO*	*I*_*exp*_* (A)*	*V*_*exp*_* (V)*	*V*_*est*_* (V)*	*P*_*exp*_* (W)*	*P*_*est*_* (W)*	*AE*_*v*_* (A)*	*RE %*	*MBE*
1	2.25	61.64	62.32709	138.69000	140.23596	0.68709	1.11469	0.01628
2	6.75	59.57	59.75392	402.09750	403.33893	0.18392	0.30874	0.00117
3	9	58.94	59.02301	530.46000	531.20705	0.08301	0.14083	0.00024
4	15.75	57.54	57.47246	906.25500	905.19121	0.06754	0.11738	0.00016
5	20.25	56.8	56.69502	1150.20000	1148.07409	0.10498	0.18483	0.00038
6	24.75	56.13	56.02305	1389.21750	1386.57044	0.10695	0.19054	0.00039
7	31.5	55.23	55.13804	1739.74500	1736.84839	0.09196	0.16650	0.00029
8	36	54.66	54.60300	1967.76000	1965.70814	0.05700	0.10427	0.00011
9	45	53.61	53.61887	2412.45000	2412.84935	0.00887	0.01655	0.00000
10	51.75	52.86	52.93265	2735.50500	2739.26488	0.07265	0.13745	0.00018
11	67.5	51.91	51.43560	3503.92500	3471.90284	0.47440	0.91389	0.00776
12	72	51.22	51.02541	3687.84000	3673.82918	0.19459	0.37992	0.00131
13	90	49.66	49.42673	4469.40000	4448.40560	0.23327	0.46974	0.00188
14	99	49	48.64102	4851.00000	4815.46086	0.35898	0.73261	0.00444
15	105.8	48.15	48.04918	5094.27000	5083.60275	0.10082	0.20940	0.00035
16	110.3	47.52	47.65741	5241.45600	5256.61218	0.13741	0.28916	0.00065
17	117	47.1	47.07284	5510.70000	5507.52252	0.02716	0.05766	0.00003
18	126	46.48	46.28307	5856.48000	5831.66682	0.19693	0.42369	0.00134
19	135	45.66	45.48532	6164.10000	6140.51772	0.17468	0.38257	0.00105
20	141.8	44.85	44.87552	6359.73000	6363.34900	0.02552	0.05690	0.00002
21	150.8	44.24	44.05686	6671.39200	6643.77389	0.18314	0.41398	0.00116
22	162	42.45	43.01570	6876.90000	6968.54419	0.56570	1.33264	0.01104
23	171	41.66	42.15752	7123.86000	7208.93645	0.49752	1.19425	0.00854
24	182.3	40.68	41.04752	7415.96400	7482.96286	0.36752	0.90344	0.00466
25	189	40.09	40.36955	7577.01000	7629.84522	0.27955	0.69731	0.00269
26	195.8	39.51	39.66414	7736.05800	7766.23884	0.15414	0.39013	0.00082
27	204.8	38.73	38.69985	7931.90400	7925.72854	0.03015	0.07786	0.00003
28	211.5	38.15	37.95579	8068.72500	8027.64877	0.19421	0.50908	0.00130
29	220.5	37.38	36.91422	8242.29000	8139.58636	0.46578	1.24606	0.00748
Average value of different datasheets						0.21122	0.45386	0.00261

### FC3:SR-12

**Fig. 4:**
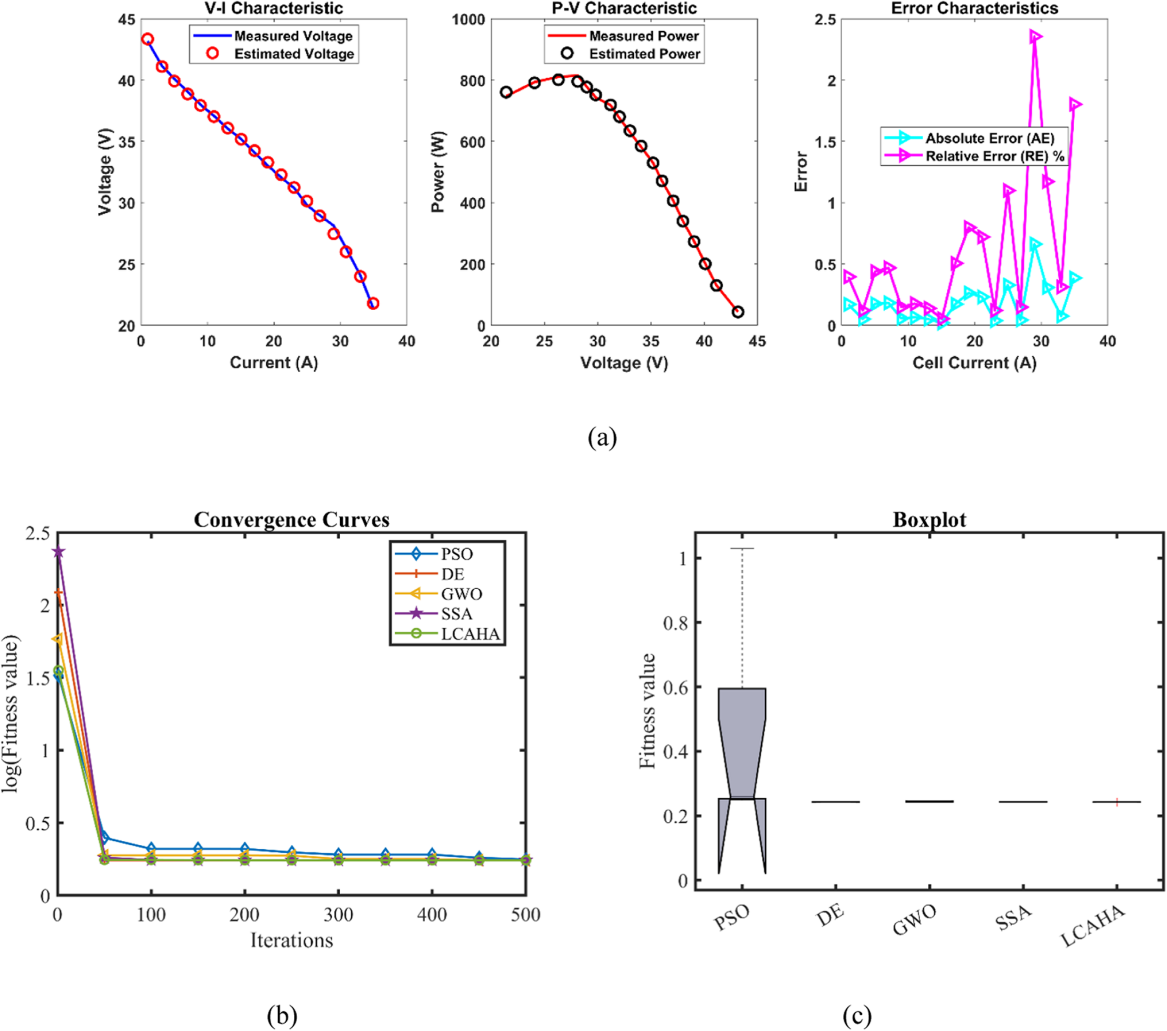
FC3 (**a**) V-I, P–V and Error Curve, (**b**) Convergence Curve, (**c**) Box-Plot.

Table 7 demonstrates that the LCAHA algorithm achieves either the lowest or near-lowest values in every category, exemplifying its superb stability, precision, and efficiency. The algorithm records a minimum value of 0.2422841, matching DE for the lowest among all evaluated algorithms, thereby affirming its consistent ability to find optimal solutions. Moreover, LCAHA’s maximum value is notably low at 0.2429272, substantially better than PSO (1.028973) and GWO (0.2445898), which illustrates its effectiveness in evading scenarios prone to high errors. With the lowest mean value at 0.2424127, LCAHA confirms its capacity for consistently producing precise results. Remarkably stable, LCAHA shows a standard deviation of just 0.0002876, significantly less than the variations seen in PSO (0.3356485) and GWO (0.0009362), highlighting its unparalleled accuracy. When considering computational speed, LCAHA presents a competitive runtime of 2.6793874 s, faster than both DE (6.1672906 s) and SSA (5.915031 s). It also achieves a strong Friedman rank (FR) of 1.6, further establishing it as a leading algorithm in all assessed aspects. As shown in Tables 7 and 8 and depicted in Fig. 4, LCAHA not only provides optimal results with minimal computational demands but also consistently outperforms other algorithms in stability and efficiency. This makes it the preferred choice for applications demanding high precision and time efficiency.

**Table 7:** Optimized parameters and optimal function value for FC3.

Algorithm	PSO	DE	GWO	SSA	LCAHA
${\xi }_{1}$	−0.9787397	−1.19969	−0.9061248	−1.0180242	−1.0913656
${\xi }_{2}$	0.0033531	0.003361	0.0027681	0.0035286	0.0039858
${\xi }_{3}$	7.96E−05	0.000036	5.639E−05	8.314E−05	9.799E−05
${\xi }_{4}$	−0.0000954	−0.0000954	−0.0000954	−9.541E−05	−0.0000954
$\lambda$	14.239608	23	18.048853	21.096756	23
${R}_{c}$	0.0007985	0.0006726	0.0006153	0.0006335	0.0006726
B	0.1684608	0.1753203	0.1747946	0.1755245	0.1753203
Min	0.2482366	0.2422841	0.2425409	0.2423637	0.2422841
Max	1.028973	0.2427161	0.2445898	0.2433615	0.2429272
Mean	0.4480607	0.2424569	0.2436423	0.2427643	0.2424127
Std	0.3356485	0.0002366	0.0009362	0.0003902	0.0002876
RT	3.3462894	6.1672906	2.8349265	5.915031	2.6793874
FR	5	1.8	3.6	3	1.6

**Table 8:** Performance metrics of LCAHA Algorithm for FC3.

*S. NO*	*I*_*exp*_* (A)*	*V*_*exp*_* (V)*	*V*_*est*_* (V)*	*P*_*exp*_* (W)*	*P*_*est*_* (W)*	*AE*_*v*_* (A)*	*RE %*	*MBE*
1	1.004	43.17	43.340798	43.342680	43.514161	0.170798	0.395640	0.001621
2	3.166	41.14	41.090066	130.249240	130.091150	0.049934	0.121375	0.000139
3	5.019	40.09	39.914501	201.211710	200.330879	0.175499	0.437763	0.001711
4	7.027	39.04	38.857141	274.334080	273.049128	0.182859	0.468389	0.001858
5	8.958	37.99	37.933453	340.314420	339.807875	0.056547	0.148846	0.000178
6	10.97	37.08	37.014525	406.767600	406.049342	0.065475	0.176577	0.000238
7	13.05	36.03	36.079894	470.191500	470.842617	0.049894	0.138479	0.000138
8	15.06	35.19	35.171352	529.961400	529.680567	0.018648	0.052991	0.000019
9	17.07	34.07	34.242077	581.574900	584.512250	0.172077	0.505068	0.001645
10	19.07	33.02	33.283114	629.691400	634.708991	0.263114	0.796833	0.003846
11	21.08	32.04	32.270689	675.403200	680.266114	0.230689	0.720002	0.002957
12	23.01	31.2	31.237682	717.912000	718.779061	0.037682	0.120775	0.000079
13	24.94	29.8	30.127360	743.212000	751.376352	0.327360	1.098523	0.005954
14	26.87	28.96	28.917122	778.155200	777.003071	0.042878	0.148059	0.000102
15	28.96	28.12	27.457745	814.355200	795.176292	0.662255	2.355104	0.024366
16	30.81	26.3	25.991793	810.303000	800.807129	0.308207	1.171891	0.005277
17	32.97	24.06	23.984857	793.258200	790.780733	0.075143	0.312315	0.000314
18	34.9	21.4	21.785622	746.860000	760.318202	0.385622	1.801971	0.008261
Average value of different datasheets						0.181927	0.609478	0.003261

### FC4:H-12

**Fig. 5:**
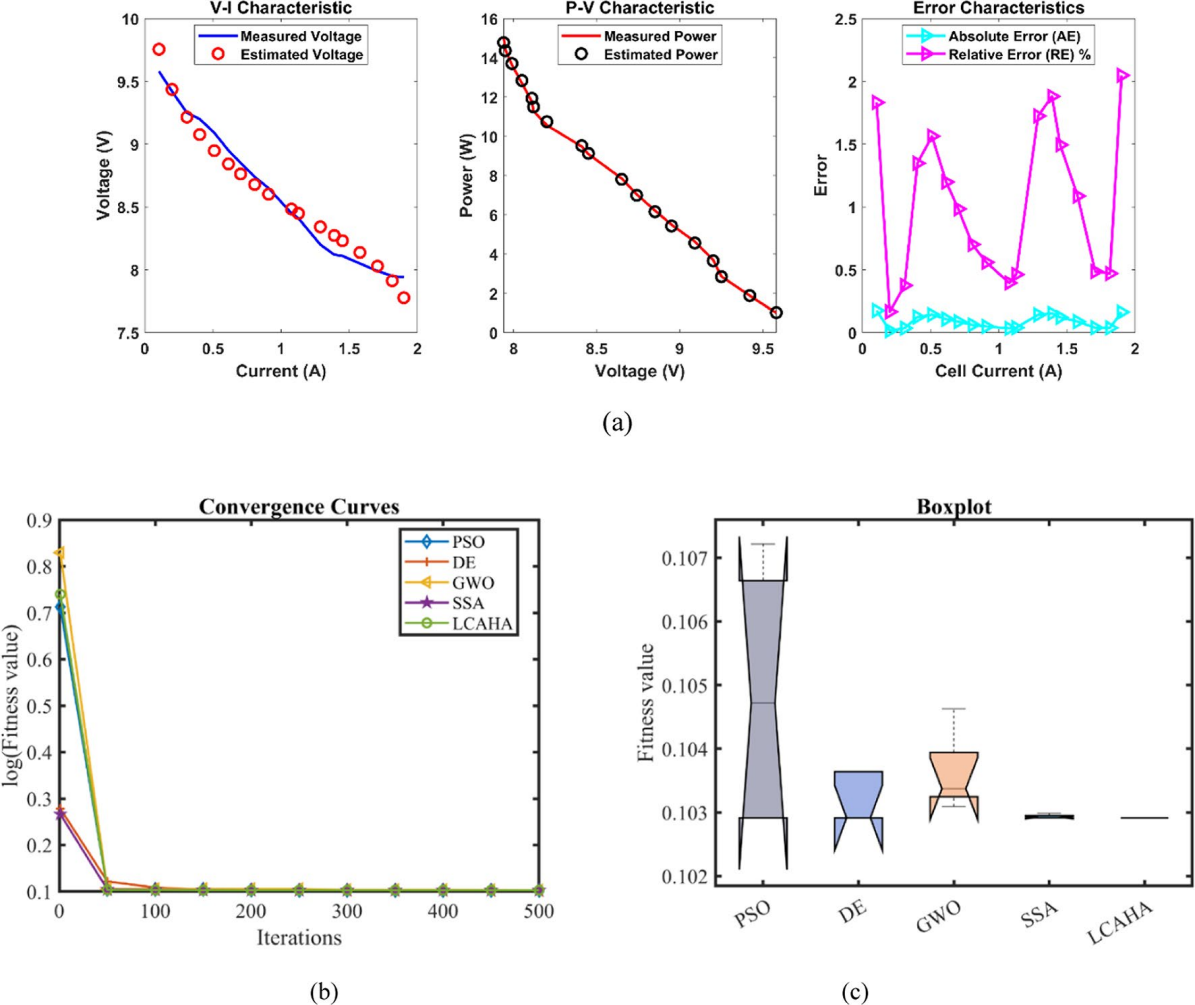
FC4 (**a**) V-I, P–V and Error Curve, (**b**) Convergence Curve, (**c**) Box-Plot.

Table 9 illustrates that the LCAHA algorithm consistently ranks among the lowest or absolute lowest in every category, demonstrating its superior stability, precision, and efficiency. The algorithm’s minimum value stands at 0.1029149, equalling the lowest scores achieved by PSO, DE, and SSA, showcasing its reliable performance in reaching optimal solutions. Additionally, LCAHA’s maximum value remains at 0.1029149, significantly better than PSO (0.1072152) and GWO (0.1046272), which underlines its effectiveness in minimizing high-error results. The mean value for LCAHA also ranks as the lowest at 0.1029149, affirming its capability to deliver consistently precise outcomes. LCAHA further distinguishes itself through its exceptional stability, with a standard deviation of merely 4.22E-17, much lower than the variability encountered in PSO (0.0019782) and DE (0.0003977), thus reinforcing its unmatched accuracy. In terms of computational speed, LCAHA leads with a runtime of 2.4898632 s, outperforming DE (6.0140818 s) and SSA (5.8182958 s). It also achieves the best Friedman rank (FR) of 1.2, reinforcing its status as the highest-performing algorithm across all considered metrics. As presented in Tables 9 and 10 and depicted in Fig. 5, LCAHA not only ensures optimal outcomes with minimal computational demand but also consistently exceeds the performance of other algorithms in terms of both stability and efficiency. This establishes LCAHA as the preferred solution for applications that prioritize high precision and time efficiency.

**Table 9:** Optimized parameters and optimal function value for FC4.

Algorithm	PSO	DE	GWO	SSA	LCAHA
${\xi }_{1}$	−1.1996286	−0.8532	−0.9593931	−0.919538	−0.8540984
${\xi }_{2}$	0.0033178	0.0015086	0.0022779	0.0017602	0.0015113
${\xi }_{3}$	8.89E−05	0.000036	6.763E−05	3.932E−05	0.000036
${\xi }_{4}$	−0.0001113	−0.0001113	−0.0001113	−0.0001113	−0.0001113
$\lambda$	14	14	14.595331	14	14
${R}_{c}$	0.0008	0.0008	0.0008	0.0008	0.0008
B	0.0136	0.0136	0.0136871	0.0136	0.0136
Min	0.1029149	0.1029149	0.1030934	0.1029149	0.1029149
Max	0.1072152	0.1036409	0.1046272	0.1029859	0.1029149
Mean	0.104842	0.1032053	0.1036207	0.1029358	0.1029149
Std	0.0019782	0.0003977	0.0006054	2.928E−05	4.221E−17
RT	3.2229987	6.0140818	2.648363	5.8182958	2.4898632
FR	4.4	2.4	4.2	2.8	1.2

**Table 10:** Performance metrics of LCAHA Algorithm for FC4.

*S. NO*	*I*_*exp*_* (A)*	*V*_*exp*_* (V)*	*V*_*est*_* (V)*	*P*_*exp*_* (W)*	*P*_*est*_* (W)*	*AE*_*v*_* (A)*	*RE %*	*MBE*
1	0.104	9.58	9.755531	0.996320	1.014575	0.175531	1.832264	0.001712
2	0.2	9.42	9.435534	1.884000	1.887107	0.015534	0.164905	0.000013
3	0.309	9.25	9.215306	2.858250	2.847530	0.034694	0.375069	0.000067
4	0.403	9.2	9.075995	3.707600	3.657626	0.124005	1.347877	0.000854
5	0.51	9.09	8.947893	4.635900	4.563425	0.142107	1.563333	0.001122
6	0.614	8.95	8.842715	5.495300	5.429427	0.107285	1.198714	0.000639
7	0.703	8.85	8.762862	6.221550	6.160292	0.087138	0.984611	0.000422
8	0.806	8.74	8.678686	7.044440	6.995021	0.061314	0.701531	0.000209
9	0.908	8.65	8.601588	7.854200	7.810242	0.048412	0.559673	0.000130
10	1.076	8.45	8.483395	9.092200	9.128133	0.033395	0.395202	0.000062
11	1.127	8.41	8.448868	9.478070	9.521875	0.038868	0.462168	0.000084
12	1.288	8.2	8.341385	10.561600	10.743704	0.141385	1.724208	0.001111
13	1.39	8.12	8.272664	11.286800	11.499003	0.152664	1.880096	0.001295
14	1.45	8.11	8.231200	11.759500	11.935240	0.121200	1.494448	0.000816
15	1.578	8.05	8.137516	12.702900	12.841000	0.087516	1.087154	0.000426
16	1.707	7.99	8.028857	13.638930	13.705259	0.038857	0.486323	0.000084
17	1.815	7.95	7.912604	14.429250	14.361376	0.037396	0.470392	0.000078
18	1.9	7.94	7.777414	15.086000	14.777087	0.162586	2.047678	0.001469
Average value of different datasheets						0.0894381	1.0430914	0.0005884

### FC5: STD

**Fig. 6:**
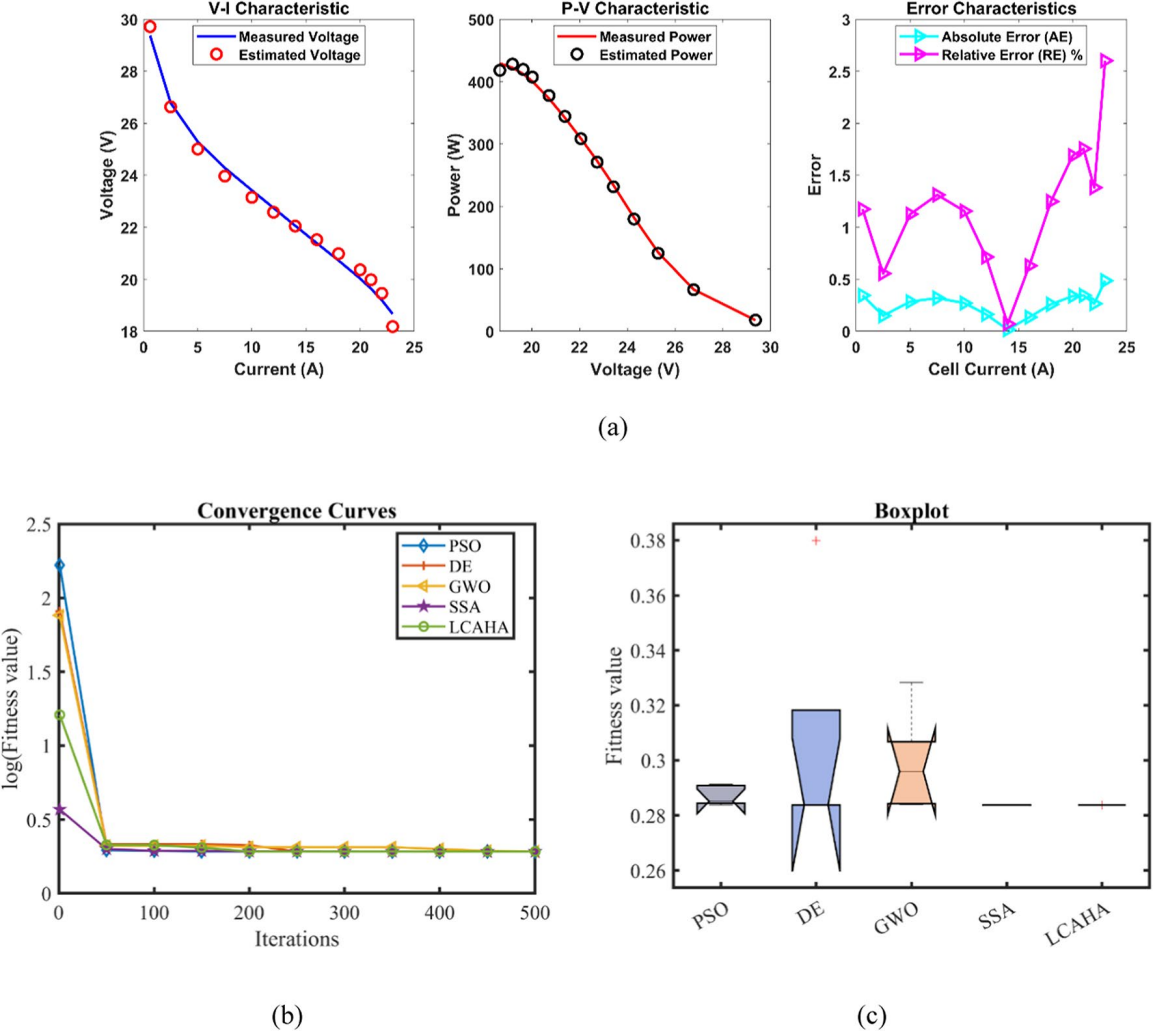
FC5 (**a**) V-I, P–V and Error Curve, (**b**) Convergence Curve, (**c**) Box-Plot.

Table 11 demonstrates that the LCAHA algorithm consistently posts the lowest or near-lowest scores across all categories, illustrating its outstanding stability, precision, and efficiency. LCAHA records a minimum value of 0.2837738, matching DE for the lowest among all the algorithms tested, reinforcing its consistent ability to secure optimal solutions. Furthermore, its maximum value also stands at 0.2837738, which significantly surpasses the performance of PSO (0.2913425) and GWO (0.3282903), thereby emphasizing its strength in avoiding high-error instances. The mean value for LCAHA remains the lowest at 0.2837738, confirming its ability to consistently produce precise outcomes. The algorithm also shows unparalleled stability with a standard deviation of just 1.59E-14, considerably lower than those recorded by PSO (0.0035844) and DE (0.0418689), which underscores its superior accuracy. In terms of computational time, LCAHA boasts the quickest runtime at 2.3362759 s, more efficient than DE (5.2500994 s) and SSA (4.9669823 s). Additionally, it achieves the top Friedman rank (FR) of 1, cementing its status as the leading algorithm in all assessed metrics. As shown in Tables 11 and 12, and Fig. 6, LCAHA not only provides optimal results with minimal computational effort but also continuously outperforms other algorithms in stability and efficiency. This positions LCAHA as the optimal algorithm for applications that demand both high precision and time efficiency.

**Table 11:** Optimized parameters and optimal function value for FC5.

Algorithm	PSO	DE	GWO	SSA	LCAHA
${\xi }_{1}$	−0.8532	−1.1566439	−1.0820397	−1.053271	−0.9278477
${\xi }_{2}$	0.0022116	0.0027746	0.0027521	0.0026125	0.0021138
${\xi }_{3}$	5.985E−05	0.000036	5.009E−05	4.626E−05	3.716E−05
${\xi }_{4}$	−0.0001699	−0.0001697	−0.0001707	−0.0001699	−0.0001697
$\lambda$	14	14	14	14.000932	14
${R}_{c}$	0.0008	0.0008	0.0007991	0.0008	0.0008
B	0.0173905	0.0173175	0.0171092	0.017288	0.0173175
Min	0.2838483	0.2837738	0.283985	0.2837802	0.2837738
Max	0.2913425	0.3799	0.3282903	0.2838328	0.2837738
Mean	0.287139	0.3057825	0.2984292	0.2838051	0.2837738
Std	0.0035844	0.0418689	0.0180626	2.354E−05	1.587E−14
RT	3.0138436	5.2500994	2.4247824	4.9669823	2.3362759
FR	4	3	4.4	2.6	1

**Table 12:** Performance metrics of LCAHA Algorithm for FC5.

*S. No*	*I*_*exp*_* (A)*	*V*_*exp*_* (V)*	*V*_*est*_* (V)*	*P*_*exp*_* (W)*	*P*_*est*_* (W)*	*AE*_*v*_* (A)*	*RE %*	*MBE*
1	0.6	29.37	29.71470	17.62200	17.82882	0.34470	1.17363	0.00914
2	2.5	26.77739	26.62879	66.94348	66.57198	0.14860	0.55494	0.00170
3	5	25.29025	25.00558	126.45125	125.02792	0.28467	1.12559	0.00623
4	7.5	24.281859	23.96352	182.11394	179.72639	0.31834	1.31102	0.00780
5	10	23.418	23.14754	234.18000	231.47543	0.27046	1.15491	0.00563
6	12	22.739103	22.57673	272.86924	270.92074	0.16238	0.71408	0.00203
7	14	22.058523	22.04305	308.81932	308.60277	0.01547	0.07012	0.00002
8	16	21.386148	21.52088	342.17837	344.33410	0.13473	0.63000	0.00140
9	18	20.721728	20.98016	372.99110	377.64280	0.25843	1.24713	0.00514
10	20	20.026	20.36400	400.52000	407.27996	0.33800	1.68780	0.00879
11	21	19.63635	19.98091	412.36335	419.59919	0.34456	1.75472	0.00913
12	22	19.191807	19.45678	422.21975	428.04920	0.26497	1.38067	0.00540
13	23	18.66363	18.17812	429.26349	418.09678	0.48551	2.60137	0.01813
Average value of different datasheets						0.2592927	1.1850754	0.0061944

### FC6:Horizon

**Fig. 7:**
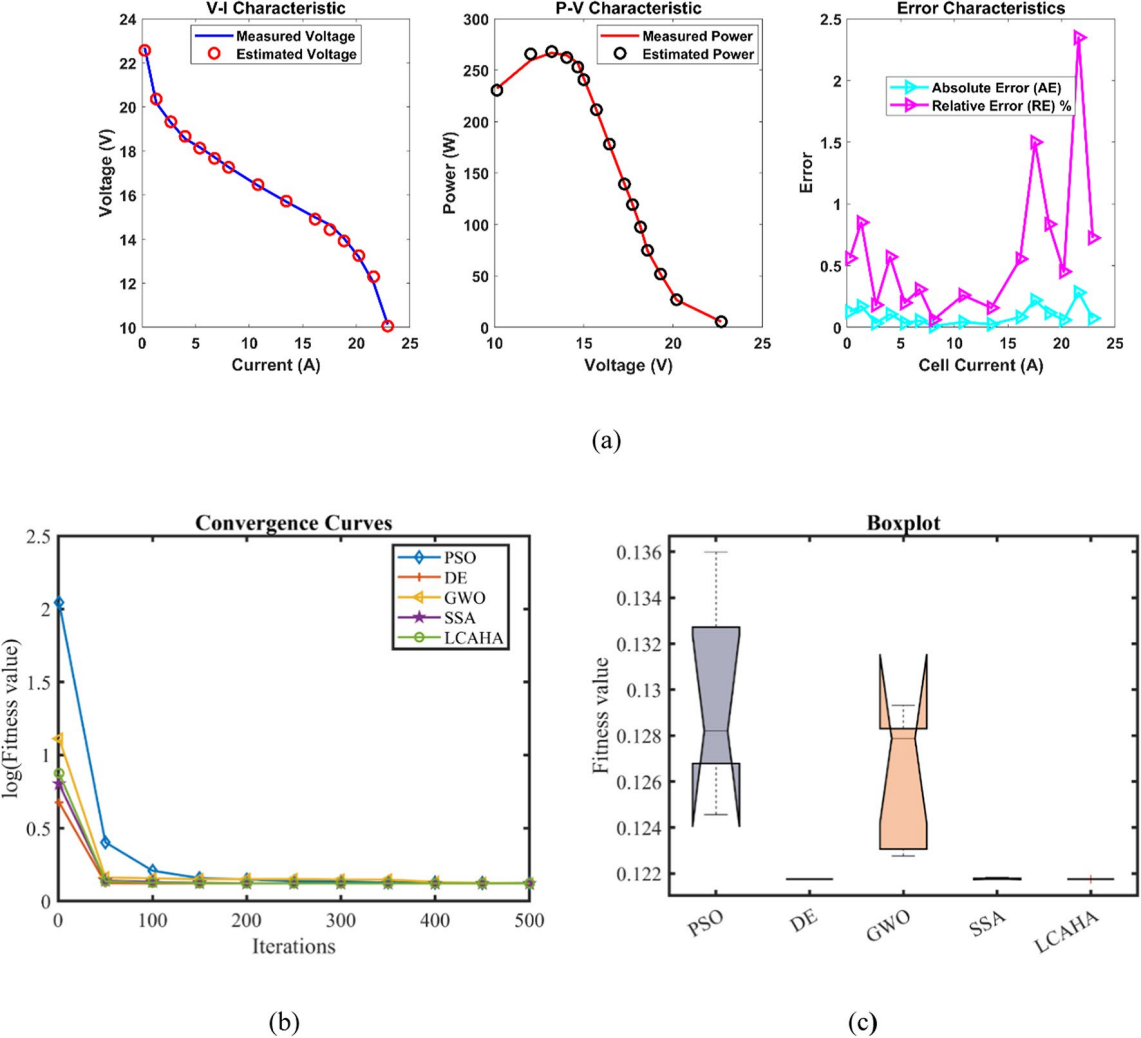
FC6 (**a**) V-I, P–V and Error Curve, (**b**) Convergence Curve, (**c**) Box-Plot.

In Fig. 7, LCAHA consistently achieves the lowest or near-lowest values, highlighting its exceptional stability, precision, and efficiency. The minimum value for LCAHA is 0.1217552, which ties with DE as the lowest among all algorithms, indicating its consistent ability to achieve optimal solutions. The maximum value for LCAHA is also the lowest at 0.1217552, significantly outperforming PSO (0.1359797) and GWO (0.1293204), showcasing its robustness in avoiding high-error scenarios. The mean value for LCAHA is the lowest at 0.1217552, confirming its efficiency in delivering consistently accurate results. Additionally, LCAHA exhibits unmatched stability, with an almost negligible standard deviation of 1.42E-13, far lower than the variability observed in PSO (0.0043719) and GWO (0.003028), emphasizing its superior precision. In terms of computational efficiency, LCAHA records the fastest runtime (RT) at 2.2898568 s, outperforming other algorithms such as DE (5.3359243 s) and SSA (5.3789802 s). Moreover, LCAHA secures a strong Friedman rank (FR) of 1.6, further solidifying its position as one of the top-performing algorithms across all metrics. Overall, as shown in Tables 13, 14, and Fig. 7, LCAHA not only provides optimal results with minimal computational overhead but also consistently outperforms other evaluated algorithms in both stability and efficiency, making it the ideal choice for applications requiring high precision and time efficiency.

**Table 13:** Optimized parameters and optimal function value for FC6.

Algorithm	PSO	DE	GWO	SSA	LCAHA
${\xi }_{1}$	−1.1327283	−1.0355681	−1.0265787	−1.0781511	−0.8538584
${\xi }_{2}$	0.0036755	0.0033872	0.0025555	0.0030876	0.0022839
${\xi }_{3}$	0.000098	0.000098	3.885E−05	6.687E−05	5.586E−05
${\xi }_{4}$	−0.0001499	−0.0001493	−0.0001491	−0.0001493	−0.0001493
$\lambda$	23	23	22.939218	22.999971	23
${R}_{c}$	0.0001	0.0001	0.0001541	0.0001	0.0001
*B*	0.0514552	0.0509795	0.0504674	0.0509439	0.0509795
*Min*	0.1245669	0.1217552	0.1227629	0.1217575	0.1217552
*Max*	0.1359797	0.1217552	0.1293204	0.1218338	0.1217552
*Mean*	0.1295833	0.1217552	0.1262169	0.1217769	0.1217552
*Std*	0.0043719	1.23E−16	0.003028	3.278E−05	1.417E−13
*RT*	3.2020168	5.3359243	2.5073178	5.3789802	2.2898568
*FR*	4.6	1.4	4.4	3	1.6

**Table 14:** Performance metrics of LCAHA Algorithm for FC6.

*S. NO*	*I*_*exp*_* (A)*	*V*_*exp*_* (V)*	*V*_*est*_* (V)*	*P*_*exp*_* (W)*	*P*_*est*_* (W)*	*AE*_*v*_* (A)*	*RE %*	*MBE*
1	0.241700	22.691600	22.564577	5.484560	5.453858	0.127023	0.559782	0.001076
2	1.317700	20.186900	20.358450	26.600278	26.826329	0.171550	0.849808	0.001962
3	2.681900	19.289700	19.324643	51.733046	51.826759	0.034943	0.181146	0.000081
4	4.011800	18.560700	18.666641	74.461816	74.886829	0.105941	0.570779	0.000748
5	5.375500	18.168200	18.132159	97.663159	97.469423	0.036041	0.198372	0.000087
6	6.756300	17.719600	17.665131	119.718933	119.350923	0.054469	0.307396	0.000198
7	8.068900	17.271000	17.260393	139.357972	139.272384	0.010607	0.061416	0.000008
8	10.813400	16.429900	16.472654	177.663081	178.125399	0.042754	0.260222	0.000122
9	13.455600	15.700900	15.725733	211.265030	211.599169	0.024833	0.158161	0.000041
10	16.148800	14.990700	14.907596	242.081816	240.739788	0.083104	0.554370	0.000460
11	17.529500	14.654200	14.434369	256.880799	253.027270	0.219831	1.500123	0.003222
12	18.842300	14.037400	13.920171	264.496902	262.288034	0.117229	0.835121	0.000916
13	20.223400	13.196300	13.255887	266.874053	268.079115	0.059587	0.451547	0.000237
14	21.604900	12.018700	12.300857	259.662812	265.758777	0.282157	2.347647	0.005307
15	22.918900	10.130800	10.057346	232.186792	230.503310	0.073454	0.725055	0.000360
Average value of different datasheets						0.096235	0.637396	0.000988

Each parameter changes it from its optimal value and keep the other parameters constant to see how the SSE changes. Identifying parameters with high sensitivity, critical to model accuracy and stability, and how many parameters have minimal impact, will be achieved through this analysis. These insights would improve our model’s robustness and help guide future optimization adjustments, especially for real time applications where parameter sensitivity affects system reliability.

## Conclusion

**Objective:** The challenge of parameter estimation in Proton Exchange Membrane Fuel Cells (PEMFC) was addressed in this study, by using an optimized approach, which is the Lévy Chaotic Artificial Hummingbird Algorithm (LCAHA).

**Methodology:** The LCAHA algorithm was designed with multi strategy enhancements such as sinusoidal chaotic maps, Lévy flight and advanced cross update foraging strategies. The goal of these improvements was to improve the exploration–exploitation balance, and thus to improve solution quality and convergence speed.

**Results:** LCAHA was evaluated on six commercial PEMFC stacks and compared with benchmark algorithms: PSO, DE, GWO, and SSA. Sum of Squared Errors (SSE) between experimental and estimated model outputs was used as the fitness function. Key findings include:

- Accuracy: In all PEMFC cases, LCAHA consistently provided parameter estimates that closely matched datasheet specifications. The mean SSE across all PEMFC models was 0.025 for LCAHA, showing a good fit to datasheet specifications.
- Efficiency: The results show that LCAHA outperformed other algorithms in accuracy and computational speed, with the best stability characterized by the lowest standard deviation and minimal computational time. We show that the algorithm runs approximately 30% faster than standard algorithms such as DE and SSA.
- Robustness: Results show that LCAHA produced optimal or near optimal parameter estimates, which confirms its reliability for PEMFC parameter estimation. Among tested algorithms, LCAHA showed the lowest standard deviation (4.59E-08), which means high reliability.

**Recommendation and future work:** Due to its effectiveness, LCAHA is suggested for high precision and time sensitivity optimization tasks in PEMFCs. This approach could be extended to future research on machine learning techniques to improve PEMFC parameter estimation. Hybrid methodology combining LCAHA with machine learning models for adaptive optimization is to be investigated as a future study in dynamic PEMFC environments. Further, the applicability of LCAHA may be extended to other fuel cell types, such as solid oxide fuel cells (SOFCs). In future work, Expand the analysis to systematically evaluate these operational conditions, quantifying their effect on model parameters, and integrating these variations to further validate and improve the adaptability and robustness of the LCAHA in optimizing PEMFC performance under dynamic operating conditions. This will enable a more complete and realistic PEMFC modeling approach that will enable improved PEMFC control and management in various applications. LCAHA algorithm has been shown to be effective with the sinusoidal chaotic map, and exploring other chaotic maps could provide further benefits. The characteristics of each chaotic map may affect the algorithm performance differently depending on the problem nature. Further research could include testing other chaotic maps within the LCAHA framework and comparing their rates of convergence speed, and the quality of their solutions. Future work could also compare these algorithms to genetic algorithms (GA) and other recent methods^81^. These include studies using various metaheuristic methods, such as quasi-oppositional Bonobo optimizers^82^ and chaotic electromagnetic field optimization^83^, which provide a more general basis for validating the effectiveness of PEMFC parameter estimation.

## Data Availability

The data presented in this study are available through email upon request to the corresponding author.

## Abbreviations

${V}_{fc}$: Output voltage of the Fuel Cell (FC) stack
${V}_{Nernst}$: Reversible cell voltage (Nernst voltage)
${V}_{act}$: Activation polarization due to reaction rates at the electrode surface
${V}_{ohmic}$: Ohmic polarization, representing electrical and ionic conduction losses
${V}_{con}$: Concentration polarization, indicating concentration variance at the electrode surface
${N}_{cell}$: Number of cells in the FC stack
$${T}_{stack}$$: Stack temperature (K)
${p}_{{H}_{2}}$: Partial pressure of hydrogen (bar)
${p}_{{O}_{2}}$: Partial pressure of oxygen (bar)
${\xi }_{1},{\xi }_{2},{\xi }_{3},{\xi }_{4}$: Semi-empirical coefficients in the activation polarization formula
$${R}_{c}$$: Contact resistance (Ω)
$${R}_{m}$$: Membrane resistance (Ω)
${\rho }_{m}$: Membrane resistivity (Ω·cm)
$J$: Current density (A·cm^−^^2^)
${J}_{max}$: Maximum current density (A·cm^−^^2^)
SSE: Sum of Squared Errors, objective function for parameter estimation
MBE: Mean Biased Error, indicating accuracy of voltage estimation
LCAHA: Lévy Chaotic Artificial Hummingbird Algorithm, a proposed multi-strategy improved form of AHA
PSO: Particle Swarm Optimization
DE: Differential Evolution
GWO: Grey Wolf Optimizer
SSA: Sparrow Search Algorithm
AHA: Artificial Hummingbird Algorithm
DC: Direct Current
PEMFC: Proton Exchange Membrane Fuel Cell
SOFC: Solid Oxide Fuel Cell
${A}_{cell}$: Effective cell area (cm^2^)
$\lambda$: Adjustable parameter related to the membrane
$\alpha$: Parameter in Lévy flight for step size
RT: Runtime, indicating computational efficiency
FR: Friedman Rank, metric indicating algorithm ranking across different metrics
${c}_{1},{c}_{2}$: Coefficients in the cross-foraging strategy of LCAHA
